# Effect of Cuprous Oxide Nanocubes and Antimony Nanorods on the Performance of Silicon Nanowire-Based Quasi-Solid-State Solar Cell

**DOI:** 10.1021/acsomega.2c04850

**Published:** 2022-12-08

**Authors:** Debanjan Maity, Partha Ghosal, Melepurath Deepa

**Affiliations:** † Department of Chemistry, Indian Institute of Technology Hyderabad, Kandi, Sangareddy, Hyderabad502284, Telangana, India; ‡ Defence Metallurgical Research Laboratory, Defence Research and Development Organization (DRDO), Hyderabad500058, Telangana, India

## Abstract

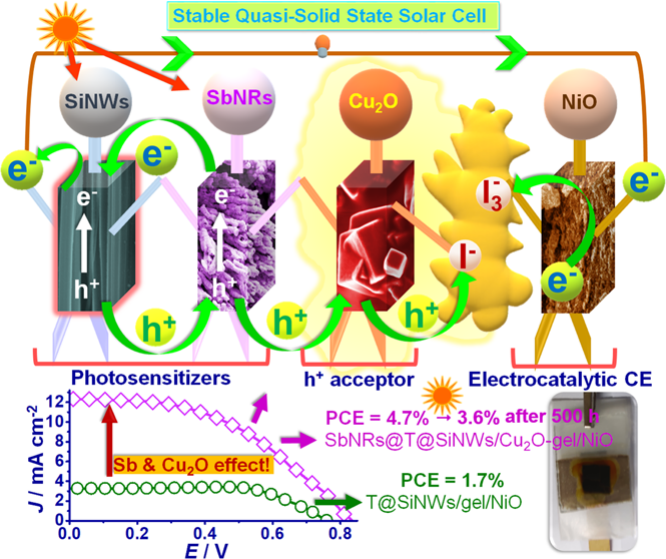

Antimony nanorods (SbNRs) anchored to vertically aligned SiNWs serve as cosensitizers and enhance the light absorption of NWs, and their favorably positioned valence band (VB) coupled with their p-type semiconducting nature allows fast hole extraction from SiNWs. Photocorrosion of SiNWs is effectively prevented by a monolayer of N-[3-(trimethoxysilyl)propyl]aniline (TMSPA). Upon assembling a quasi-solid-state solar cell with a SbNRs@TMSPA@SiNW photoanode, a triiodide–iodide (I_3_^–^/I^–^) redox couple-based gel encompassing dispersed p-type cuprous oxide nanocubes (Cu_2_O NCs) as the hole transport material. and an electrocatalytic NiO as the counter electrode, a power conversion efficiency (PCE) of 4.7% (under 1 sun) is achieved, which is greater by 177% relative to an analogous cell devoid of the Cu_2_O NCs and SbNRs. SbNRs at the photoanode maximize charge separation and suppress electron–hole and electron–I_3_^–^ recombination at the photoanode/electrolyte interface, thereby improving the overall current collection efficiency. Concurrently, the Cu_2_O NCs facilitate hole scavenging from SbNRs or SiNWs and relay them rapidly to the I^–^ ions in the electrolyte. Optically transparent and mesoporous NiO with a VB conducive to accepting electrons from FTO permits abundant interaction with I_3_^–^ ions. The high PCE is a cumulative outcome of the synergistic attributes of SbNRs, Cu_2_O NCs, and NiO. The SbNRs@TMSPA@SiNWs/Cu_2_O-gel/NiO solar cell also exhibits a noteworthy operational stability, for it endures 500 h of continuous 1 sun illumination accompanied by an ∼24.4% drop in its PCE. The solar cell architecture in view of the judiciously chosen components with favorable energy level offsets, semiconducting/photoactive properties, and remarkable stability opens up pathways to adapt these materials to other solar cells as well.

## Introduction

Silicon nanowires (SiNWs) are promising photoanode scaffolds in liquid junction solar cells (LJSCs), wherein the vertically aligned nanowires with high surface areas trap the maximum possible visible light for conversion, resulting in high power conversion efficiencies (PCEs) in the range of 6–13%.^1−5^ One of the most attractive features of SiNWs is their one-dimensional structure, which allows radial junction hole transport and transfer, and other pertinent properties include low reflectivity (less than 5% in the visible region),^6−8^ synthetic ease via metal-catalyzed n-Si wafer etching, and the ability of the SiNWs to deliver high PCEs, despite surface defects and impurities, a property that planar-Si solar cells cannot boast of.^9,10^ The simplest configuration of an LJSC comprises a photoanode of SiNWs grown directly over a n-Si wafer in direct contact with a hole transport layer (HTL), with the most widely used one being an aqueous solution of HBr and Br_2_, exposed to an electrocatalytic counter electrode (CE), usually Pt on the other side.^11^ However, this cell, SiNWs/HBr,Br_2_/Pt mesh, having a PCE of 0.29%^11^ suffers from several drawbacks, the principal one being the extremely high corrosivity of the acid electrolyte, which chemically erodes the SiNWs leading to the rapid decline in the cell performance within a few hours.^2^ Over the years, this architecture has been modified for both improved PCE and stability. These approaches include the anchoring of catalytic Pt or Au metal nanoparticles to SiNWs^1^ by coating SiNWs with a layer of carbon to protect them from the electrolyte attack during cell operation and then decorating them with Pt NPs for improved charge separation ensuing in a record PCE of 10.86%.^3^

Some interesting configurations that replaced the corrosive Br_2_/Br^–^ HTL with the conducting polymer, poly(3,4-ethylenedioxythiophene):poly(4-styrene sulfonate) (PEDOT:PSS), and still retained reasonably high efficiencies of ∼6.9% with tapered SiNW,^12^ 9.3% with optimal annealing and length conditions,^13^ and 8.4% with short SiNW lengths of ∼0.4 μm and good coverage of PEDOT:PSS,^14^ have been reported. Yoon et al. reported a hierarchical Si- and PEDOT:PSS-based hybrid solar cell having a siloxane interlayer. The device showed a very high efficiency of 17.34%.^15^ Zhang et al. reported the use of a composite of polyethylenimine and cesium carbonate as an interlayer between the nanostructured silicon and Al metal at the rear side of the device. Cs_2_CO_3_ served as an electron injection layer and thermal evaporation of Al on top, which resulted in the formation of Al–O–Cs bonds, which lowered the work function of Al. The device showed a PCE of 13.7%.^16^ Shen et al. used copper thiocyanate and PEDOT:PSS on SiNWs as a double hole transport layer. The tetramethylammonium hydroxide-treated SiNW absorber-based device showed a PCE of 12.24%.^17^ Zhang et al. reported a hybrid Si solar cell having a methyl/allyl organic monolayer as a surface passivating agent. The device delivered a PCE of 10.2%.^18^ Pudasaini et al. used ultrathin atomic layer deposition (ALD)-grown Al_2_O_3_ as a surface passivating layer on Si nanopillars for a hybrid solar cell. The device gave a PCE of 10.56% efficiency.^19^ Wang et al. showed that a thin SiO_2_ coating on SiNWs can act as a surface passivating layer and the cell produced a PCE of 12.4%.^20^

Yet, another heterojunction structure of ITO/V_2_O_5_/n-SiNWs/TiO_2_/Al, wherein V_2_O_5_ and TiO_2_ serve as the hole- and the electron-selective layers, delivered a PCE of 12.7% due to the light trapping effect of the wires and the excellent carrier transport due to the oxides.^21^ Another notable report involved the covalent bonding of diallyl disulfide to the SiNWs via UV irradiance, imparting good air stability and PCE (7.2%) to the cell.^22^ Some of our earlier configurations comprised of decorating SiNWs with hole-transporting nanostructures such as Se NPs (7%), C@Te nanorods (11.5%), and graphene quantum dots or GQDs (13.2%).^4,5,23^ However, these cells employed the HBr, Br_2_ electrolyte, thus limiting their practical applications, with some improvement in stability achieved subsequently, by the use of I_3_^–^/I^–^ electrolytes at the cost of cell efficiency.^24,25^ In view of the above developments and lacunae, this report expounds the fabrication of a stable and an efficient solar cell, with photoactive and hole-conducting antimony nanorod (SbNR)-decorated [3-(trimethoxysilyl)propyl]aniline (TMSPA)-passivated SiNWs as the photoanode, an I_3_^–^,I^–^ gel enriched with Cu_2_O nanocrystals as the HTL, and with a NiO CE. The highlights of this novel solar cell are (1) the low cost, ease of availability, and low toxicity of the elements (Sb, Ni, and Cu) in the photo/electroactive materials involved, (2) the ingenious approach of dispersing Cu_2_O in the gel to improve hole transfer and transport via favorable energy level offsets, (3) the application of the highly electrocatalytic and stable NiO CE film for maximum charge separation and therefore efficiency, and (4) the high stability induced by the TMSPA self-assembled monolayer that passivates the surface suppressing the rigorous oxidation of SiNWs in the electrolyte.^26^ Contrasting with earlier studies, where costly Pt or Au NPs are used in the photoanode, and the expensive and opaque Pt mesh is used as the CE,^3^ that too in an O-ring-type cell, thus restricting the cell’s practical application; here, a quasi-solid-state cell is fabricated with an optically transparent NiO/FTO CE, thus allowing direct solar light penetration from the front side, enabling its real-world utilization. This unique inexpensive, innovative, stable cell architecture of SbNRs@TMSPA@SiNWs/Cu_2_O-I_3_^–^,I^–^ gel/NiO@FTO opens up future possibilities for easily doable interface engineering via chemical methods for SiNWs with potential for scale-up and commercial application as well.

## Experimental Section

### Chemicals

n-Type silicon wafers (CZ, 1–10 Ω cm) were purchased from Siegert Wafer GmbH, Germany. Fluorine-doped tin oxide (FTO) with a sheet resistance of ∼25 Ω cm^2^ was purchased from Pilkington. Antimony chloride (SbCl_3_), zinc (Zn) dust, copper nitrate trihydrate (Cu(NO_3_)_2_·3H_2_O), silver nitrate (AgNO_3_), nickel nitrate hexahydrate (Ni(NO_3_)_2_·6H_2_O), hexamethylenetetramine or hexamine (C_6_H_12_N_4_), lithium iodide (LiI), iodine (I_2_), poly(ethylene oxide) (PEO, MW: 600 000), N-[3-(trimethoxysilyl)propyl]aniline (TMSPA), 3-propyl-1-methyl imidazolium iodide (PMII), 1-ethyl-3-methyl imidazolium thiocyanate (EMISCN), hydrofluoric acid (HF, 40%), ammonia (NH_3_, 32%), hydrogen peroxide (H_2_O_2_, 30%), toluene (C_7_H_8_), formic acid (H_2_CO_2_, 95%), hydrochloric acid (HCl, 37%), sulfuric acid (H_2_SO_4_, 98%), ethanol, acetone, and isopropanol were purchased from Merck. Ultrapure water with a resistivity of ∼18.2 MΩ cm was obtained from the Millipore Direct-Q3 UV system.

### Etching of Silicon Wafer

The silicon wafers were cut into 1.5 × 1 cm^2^ pieces, which were cleaned sequentially in acetone and deionized water and added to the boiling Piranha solution (H_2_SO_4_/H_2_O_2_ = 3:1 v/v) to remove unwanted organic residues. After the cleaning process, the wafers were rinsed in deionized water and dipped in 5% HF solution to remove the SiO_2_ layer from the surface. The etching was performed in a plastic beaker containing an aqueous mixture of 5 mol/L of HF and 0.02 mol/L of AgNO_3_ for 15 min. The silver-coated etched silicon wafers were dipped in a solution of NH_3_ and H_2_O_2_ having a 3:1 ratio to remove the silver coating. Black-colored etched silicon wafers were cleaned in distilled water and dried at room temperature and were ready to use.^27^

### Synthesis of Sb Nanorods and Cu_2_O Nanocubes

Antimony (Sb) nanorods were synthesized using a solvothermal method by following a previous report.^28^ SbCl_3_ (521 mg) was dispersed in 40 mL of toluene by vigorous stirring at 5000 rpm for 30 min and transferred to a 50 mL Teflon-lined stainless steel autoclave. Zn powder (75 mg) was added to the solution, and the autoclave was placed in a vacuum oven at 180 °C for 12 h. The autoclave was allowed to cool for a further 6 h, the black precipitate was filtered, and the residue was washed thoroughly using absolute alcohol, dilute HCl, and deionized water sequentially. Finally, the product was dried at 50 °C under vacuum and collected.

Cuprous oxide nanocubes (NCs, p-type) were synthesized using a hydrothermal method:^29^ formic acid (4 mL) was added to a 0.05 M ethanolic solution (60 mL) of copper nitrate and ultrasonicated for 10 min. The solution was transferred to a Teflon-lined stainless steel autoclave (100 mL), sealed, and kept in an oven at 150 °C for 2 h, cooled thereafter. A brown-colored product was collected, washed in ethanol and distilled water several times, and dried at 60 °C for 5 h.

### Fabrication of NiO Counter Electrode (CE)

FTO (SnO_2_:F)-coated glass substrates were cleaned sequentially in detergent, acetone, IPA, and deionized water. Optically transparent NiO counter electrodes were fabricated by a potentiostatic method^30^ in an aqueous solution of 0.05 M hexamine, 0.05 M Ni(NO_3_)_2_, and 0.05 (M) KCl in a three-electrode system having FTO-coated glass as the working electrode, Pt rod as the counter electrode, and Ag/AgCl/KCl as the reference electrode. A constant dc potential of −1.1 V was applied for 600 s, and a light green-colored Ni(OH)_2_ compound was grown over FTO. Ni(OH)_2_-coated FTO electrodes were annealed at 350 °C to obtain a brown-colored NiO electrode.

### Solar Cell Fabrication

The surface of the SiNWs was passivated by dipping etched SiNW wafers in a 0.05 M ethanolic solution of TMSPA (N-[3-(trimethoxysilyl)propyl] aniline) for 24 h, dried at 60 °C, and TMSPA-coated etched wafers were obtained. SbNRs were dispersed in isopropanol by ultrasonication for 1 h. The drop volume and concentration of the SbNR dispersion in isopropanol are 100 μL and 1 mg/mL, respectively. The dispersion was drop-cast over SiNWs, dried at 60 °C, and used as a photoanode. A homogeneous liquid electrolyte containing 0.01 M I_2_, 0.02 M LiI, PMII (3.25 mL), and EMISCN ionic liquid (1.75 mL) in PC (20 mL) was prepared. The gel electrolyte was prepared by adding 5% (W/V) PEO to the electrolyte without iodine under vigorous stirring. The gel formation took place at 60 °C after 15 min of addition of PEO to the electrolyte. The hot gel electrolyte was allowed to cool down, and then iodine was added and mixed to form a uniform iodine–iodide-based gel electrolyte. A composite electrolyte was prepared by dispersing Cu_2_O NCs in the iodide-based liquid electrolyte along with PEO. A thick parafilm separator with a cavity size of 10 mm × 5 mm was placed over the NiO@FTO CE, and the gel electrolyte was applied into the cavity. SbNRs@TMSPA@SiNW electrode was placed on it. Electrical contact from the photoanode was taken through a copper wire/Ag paste on the back side of the photoanode. The device fabrication is shown in detail in Scheme S1 using cartoons and photographs.

### Instrumental Methods

X-ray diffraction patterns of the materials were recorded on a PANalytical X’PertPRO instrument with Cu Kα (λ = 1.5406 Å). The morphology of the materials was studied on a field emission scanning electron microscopy instrument (Zeiss Cross beam 350 FEG-SEM with LASER-FIB). The UV–visible absorption spectra of the nanomaterials were recorded in the diffuse reflectance mode and converted to absorption using the Kubelka–Munk function on a UV–vis spectrophotometer equipped with an integrating sphere. Fluorescence spectra were collected on a Horiba Fluoromax-4 spectrophotometer. The transmission electron microscopy (TEM) images of Cu_2_O NCs and SbNRs were recorded on a JEOL 2100 microscope working at an accelerating voltage of 200 kV. Linear sweep voltammetry, cyclic voltammetry, chronoamperometry, and electrochemical impedance spectra (EIS) of the cells were studied on an Autolab PGSTAT 302N connected to a frequency response analyzer (FRA) and NOVA 1.11 software under an ac amplitude of 20 mV over the frequency range of 1 MHz to 0.01 Hz. *I*–*V* measurements of solar cells were recorded on a Newport Oriel 3A solar simulator with a Keithley model 2420 source meter. A 450 W xenon arc lamp was used as a light source with a light intensity of 100 mW cm^–2^ and of Air Mass (AM) 1.5 G illumination; the spatial uniformity of irradiance was determined by calibrating with a 2 cm × 2 cm Si reference cell traceable to NREL and reaffirmed with a Newport power meter. EQE versus wavelength spectra were recorded for the LJSCs over a wavelength range of 360–1040 nm on a quantum efficiency measurement system, an Oriel IQE-200 compliant with ASTM E1021-06. A 250 W quartz tungsten halogen lamp functions as the light source, the monochromator path length was 1/8 m, and the spot size was 1 mm × 2.5 mm rectangular at focus.

## Results and Discussion

### Structures of SbNRs and SiNWs

**Scheme 1:**
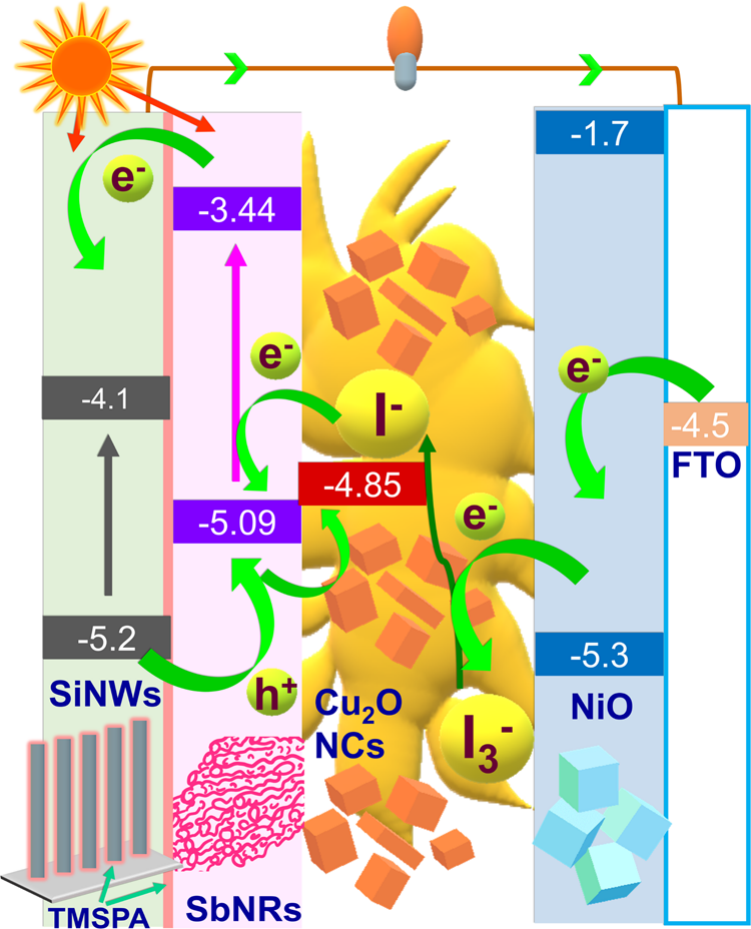
Energy Band Diagram of the SbNRs@TMSPA@SiNWs/Cu_2_O-Gel/NiO Cell Architecture

Prior to analyzing the structure, the energetics of the full solar cell, SiNWs/TMSPA/SbNRs/Cu_2_O-I_3_^–^-I^–^/NiO/FTO, is discussed and the energy band diagram is shown in Scheme 1. At the photoanode, the photogenerated electrons from both SiNWs and SbNRs reach the CE, where I_2_ or I_3_^–^ species reduce to I^–^ at the NiO/electrolyte interface. I^–^ ions then diffuse to the photoanode, get oxidized, and regenerate the SiNWs and SbNRs. Concurrently, Cu_2_O NCs, which are dispersed in the electrolyte, facilitate hole transfer and transport, owing to their p-type conducting nature and their favorably positioned Fermi level (*E*_F_) at −4.5 eV.^31^ Hole transfer occurs in the following direction: from the VB of SiNWs to the VB of SbNRs and then to I^–^ via the *E*_F_ of Cu_2_O or directly to I^–^ ensuring circuit closure. All valence band/conduction band (VB/CB) positions were determined from CV plots (Figure S1 and Table S1) and optical band gaps.

**Figure 1:**
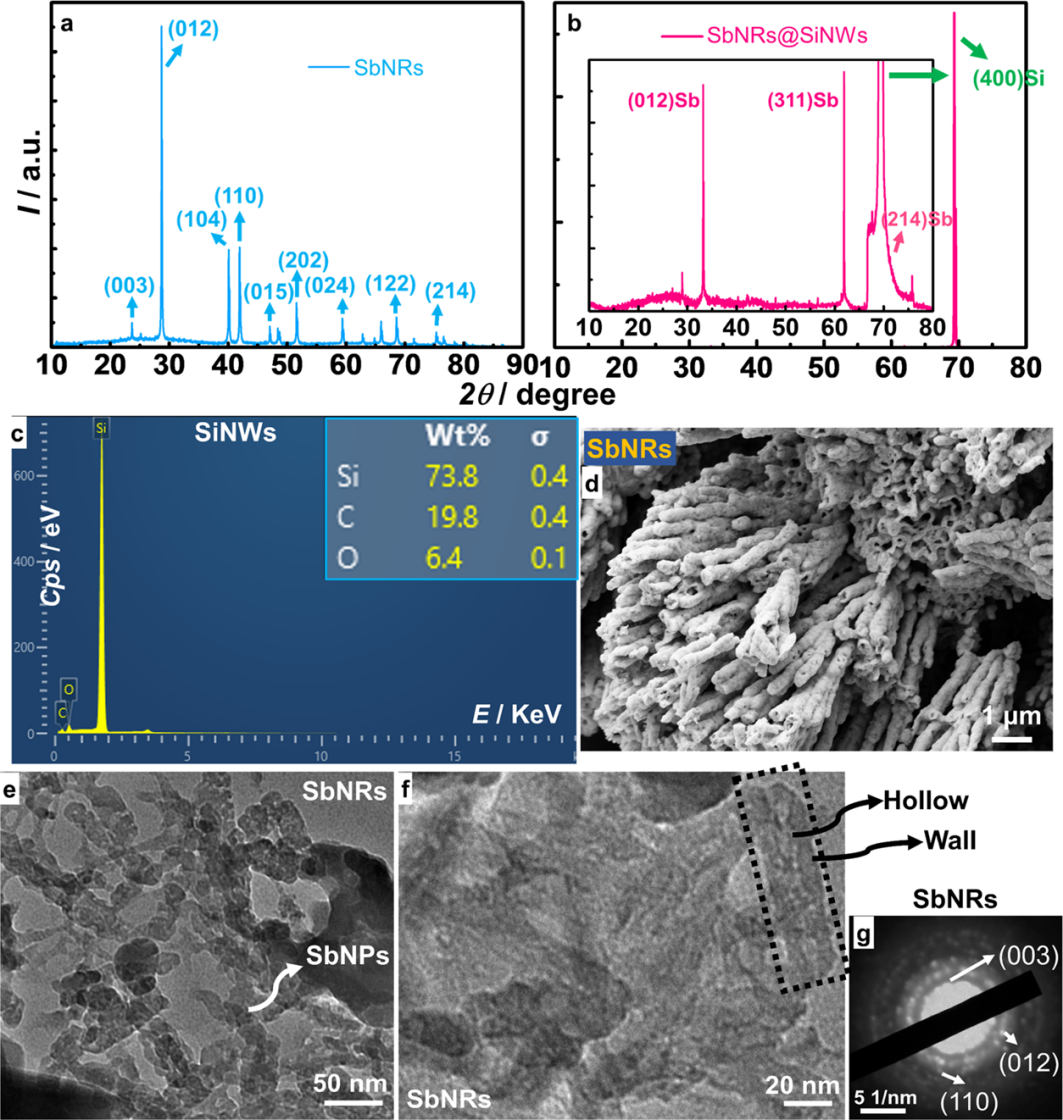
XRD of (a) SbNRs and (b) SiNWs (the inset shows the pattern for SbNRs@SiNWs). (c) EDX analysis of SiNWs. (d) FE-SEM image of SbNRs. (e, f) TEM images and (g) SAED pattern of SbNRs.

The XRD pattern of Sb nanorods (Figure 1a) shows multiple peaks located at 2θ = 23.59, 28.64, 40.06, 41.89, 47.03, 51.59, 59.44, 68.83, and 76.67° corresponding to the (003), (012), (104), (110), (015), (202), (024), (122), and (214) planes, respectively, of the rhombohedral crystal structure with *a* = *b* = 4.3 Å and *c* = 11.2 Å aligning with JCPDS 85-1324. A preferential growth for SbNRs along the (012) plane that represents the radial direction of the nanorod is evidenced by this peak being the most intense one among all observed peaks. The data confirms the formation of a pristine crystalline Sb phase, with no impurities, implying that complete reduction of SbCl_3_ is accomplished by the reductive action of metallic Zn powder in the solution phase under solvothermal conditions and follows the equation furnished below
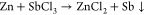
1

Pure SiNWs (Figure 1b) show an extremely strong and intense reflection at 2θ = 69.5°, which is assigned to the (400) plane of highly crystalline and well-oriented SiNWs with a face-centered cubic (fcc) lattice structure as per JCPDS: 892955. The inset displays the pattern obtained for Sb nanorods deposited over SiNWs with two sharp peaks at 2θ = 33.21 and 61.87°, which arise from the (012) and (311) planes of the rhombohedral phase of Sb and a third peak at 69.5° that originates from the (400) plane of SiNWs. The (400) plane has a broad base with a shoulder at 71° stemming from the (214) plane of Sb, thus confirming the formation of the composite. The formation of SiNWs entails the oxidation Si surface, for it involves the chemical etching of Si. This is also accompanied by the formation of Si–O–O–Si-, Si–OH-, and Si–O–Si-type covalent bonds on the surface of SiNWs. The oxygen functionalities and Sb interact via van der Waals attractive forces, which possibly causes these peak shifts for Sb. The diffuse reflectance spectra in Figure S2 show that the average reflectances of planar Si and SiNWs are ∼55 and ∼5%, respectively. This lowering in the reflectance of SiNWs is due to the light trapping property of vertically grown nanowires.

FE-SEM images of SiNWs (Figure S3, Supporting Information) reveal vertically aligned nanowires, which are slightly tilted in some regions; the wires are densely packed and have diameters in the range of 100–200 nm and 3–7 μm in lengths. The EDAX plot (Figure 1c) shows distinct signals due to Si, C, and O, and their elemental proportions are 73.8, 19.8, and 6.4%. The surface of SiNWs is oxidized to some extent upon exposure to an ambient atmosphere, which is rich in dissolved oxygen and moisture, and this contributes to the oxygen signal. Cross-sectional FE-SEM image of SbNRs (Figure 1d) exhibits clusters of juxtaposed elongated structures, with lengths and diameters in the ranges of 1–3 μm and 100–350 nm, respectively. The rods have a rough texture, appear to be hollow and open-ended, and the average thickness of the walls is approximately 15 nm. Similar hollow Sb nanotubes have been reported by Li et al.^28^ The solvent toluene functions as the structure directing agent and first induces the formation of lamellar layers of covalently bonded Sb atoms, which then roll up under solvothermal conditions to form the rods. The Zn^2+^ ions and solvent molecules are adsorbed on the layers, and they cumulatively provide the driving force for the formation of rodlike shapes. To minimize the surface energy and the thermal stresses generated during the heat treatment, the Sb layers curl up and form these shapes.^28^ The high-resolution TEM images of SbNRs (Figure 1e) show the rodlike structures to be composed of Sb nanoparticles, 10–40 nm in dimensions, almost fused to each other with indistinct grain boundaries and their hollow structure is evident in Figure 1f. Figure 1g displays the associated selected area electron diffraction (SAED) pattern; it is composed of closely spaced bright spots superimposed over concentric diffuse rings, and the spots are indexed to the (003), (012), and (202) planes of rhombohedral Sb.

**Figure 2:**
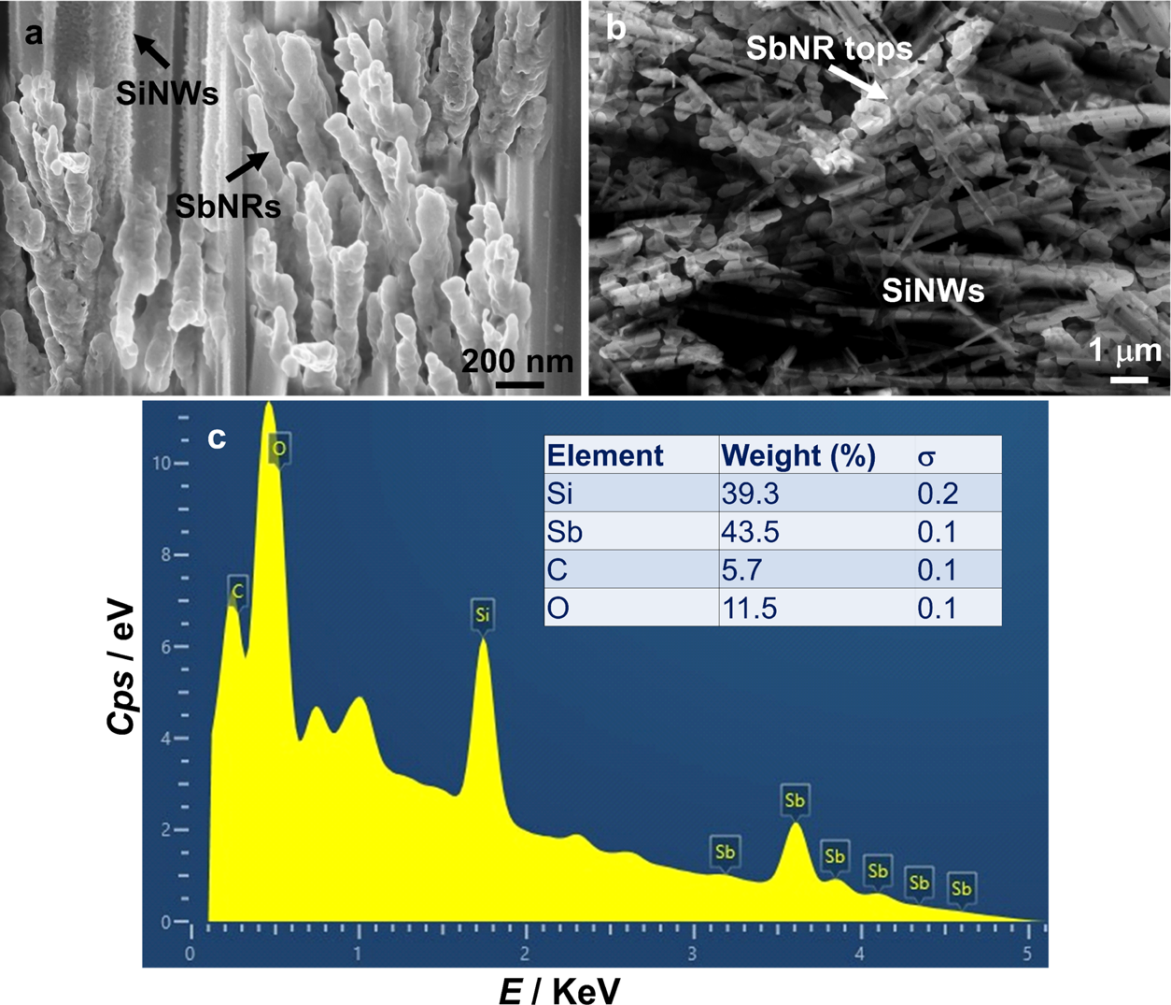
(a) Cross-sectional FE-SEM image and (b) top view of SbNR-decorated SiNWs. (c) EDX analysis of the top surface of SbNRs@SiNWs.

FE-SEM image (side-view) of SbNRs@SiNWs is shown in Figure 2a, wherein clusters of SbNRs are seen to be wedged in the gaps between the SiNWs. The top-view image (Figure 2b) shows the Sb nanorod-tops and they appear to be deposited over the SiNWs. The EDAX plot extracted from the top surface of the composite electrode (Figure 2c) reveals the Si and Sb proportions to be nearly the same, 39.3 and 43.5%, with multiple strong signals emanating from elemental Sb, thereby implying that the SiNWs are well coated with SbNRs.

### Absorbance, Fluorescence, and Energetics of Photoanode Components

**Figure 3:**
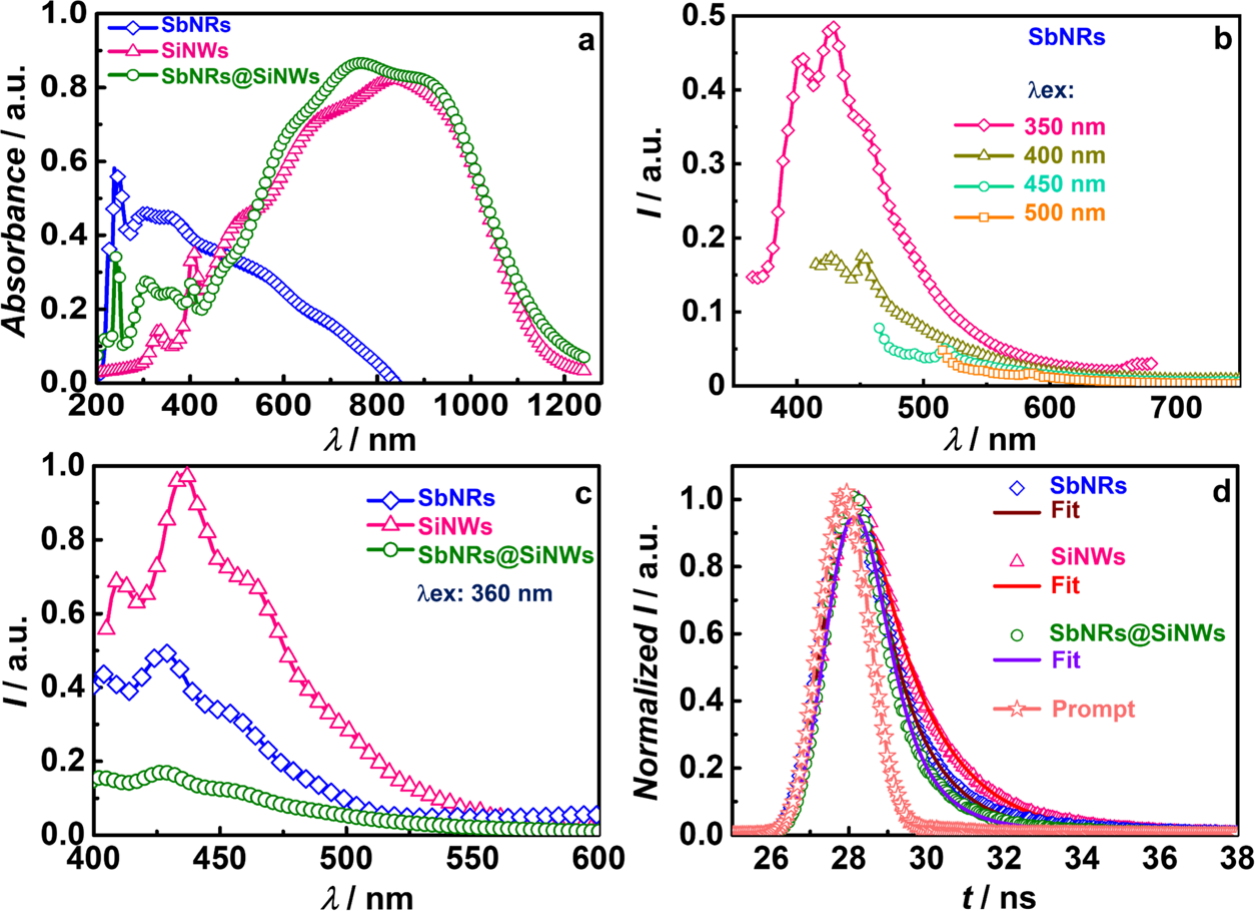
(a) Absorption spectra of SbNRs, SiNWs, and SbNRs@SiNWs. Fluorescence spectra of (b) SbNRs at λ_ex_ = 350, 400, 450, and 500 nm and (c) SbNRs, SiNWs, and SbNRs@SiNWs at λ_ex_ = 360 nm. (d) Time-resolved fluorescence decay traces of SbNRs, SiNWs, and SbNRs@SiNWs and colloidal starch solution (prompt).

The UV–visible absorption spectra of SbNRs, SiNWs, and SbNRs@SiNWs are displayed in Figure 3a. SbNRs are highly absorbing in the visible region, for they show a sharp peak with λ_max_ at 250 nm, followed by a broad absorption band spanning from ∼300 to 800 nm. A similar broad and rather flat visible absorption band was observed in the past for SbNPs synthesized in solution phase using NaBH_4_ as the reducing agent.^32^ The corresponding Tauc plot (Figure S4b) of (α*h*ν)^2^ versus *h*ν exhibits a linear dependence over 1.8–3.2 eV and follows the relation: (α*h*ν)^2^ = *E*_g_ – *h*ν, which is valid for direct gap semiconductors. Consequently, in SbNRs, the transitions from the VB to the conduction band CB are direct, as shown in the inset of Figure S4b. The intercept on the abscissa represents *E*_g_, the band gap of SbNRs, and it is found to be 1.65 eV. Pure SiNWs show a distinct broad peak in the visible region, which tapers off in the NIR region, and from the associated Tauc plot (Figure S4a), the band gap is estimated to be 1.1 eV. The SbNRs@SiNW composite’s absorption profile represents a combination of the individual absorption features of the two components. The HOMO position of SbNRs is obtained from cyclic voltammetry (Figure S1 and Table S1, Supporting Information).

SbNRs were also found to be highly luminescent, and fluorescence spectra recorded at different excitation wavelengths of 350, 400, 450, and 500 nm are shown in Figure 3b. The quantum yield of SbNRs was found to be 0.41, and it was measured using a Rhodamine 6G dye solution as a reference (Figure S5). More details are provided in the Supporting Information. Under λ_ex_ = 350 nm, twin peaks at 403 and 427 nm followed by a shoulder at 454 nm were obtained; the intensity decreases and the peaks undergo a red shift at λ_ex_ = 400 nm and the profiles are rather featureless under still longer excitation wavelengths. Since the band gap of SbNRs is 1.65 eV, and the observed emission peaks are observed at energies greater than the band gap, these peaks are attributed to excitation to higher energy levels in the CB followed by radiative transition to the VB maximum.

Excitation wavelength-dependent fluorescence is usually caused by the presence of polar groups in the fluorophore, like graphene oxide (GO) nanosheets, which typically have a high proportion of −COOH groups and the use of a polar solvent like water or alcohol.^33^ It is very natural for polar −OH groups to be easily adsorbed on the surface of SbNRs during the final washing with ethanol/dilute acid/water media. Furthermore, the SbNRs, like GO, are also dispersed in a polar medium like isopropanol. The giant red-edge effect, i.e., the shift of emission maxima to longer wavelengths, in materials like GO in a polar solvent is ascribed to a slow solvent relaxation process, caused by the local chemical environment of the material, and considering the similarity of the role played by the surface groups, and the dispersion medium, the same reason can be assigned to this case as well.

The defect states in SiNWs play a crucial role in controlling the device performance. To understand more about defects, the fluorescence spectrum of SiNWs was measured under an excitation wavelength of 360 nm (Figure 3c) and it showed a broad emission peak in the wavelength range of 400–600 nm. This peak lies over an energy range that is greater than the band gap of SiNWs, which is 1.1 eV. This higher energy emission is therefore due to the Si–O-based defects; these are surface defects formed by the oxidation of the SiNW surfaces. Contrary to planar Si, SiNWs are characterized by a significantly enhanced surface-to-volume ratio and this allows easy diffusion and adsorption of moisture and oxygen deep in between the wires, thus causing the oxidation of SiNW surfaces. The formation of Si–O–H, Si–O–O–Si, and Si–O–Si bonds occurs at the surfaces, and the energy levels of these defects lie at values that are positioned above the conduction band minimum, which explains the higher energy electronic transitions and hence the emission in the visible region. Similar observations have been made in the past for 1D-SiNWs prepared without a catalyst.^34^ In another study on SiNWs prepared by laser ablation at high temperature, the emission in the visible region was attributed to the defect states produced by the amorphous silicon oxide layer formed over crystalline SiNWs.^35^ More experimental evidence for the presence of defect states was obtained through the deconvolution of the core level Si2p XPS spectrum, shown in the Supporting Information (as Figure S6). It shows a distinct Si–O signal at 104 eV from the oxidized Si surface besides the spin-orbital components of Si2p_3/2_ and Si2p_1/2_ at 99.5 and 103 eV, respectively. The intensities are 8.14, 74.27, and 17.59%. The relatively good intensity of the Si–O peak indicates that defects are fairly prominent. These defects are passivated to some extent by coating TMSPA.

The composite shows a reduced fluorescence intensity compared to pristine SiNWs due to thermodynamically allowed excited electron injection from the CB of SbNRs (at −3.1 eV) to the CB (at −4.1 eV) of SiNWs. This is also depicted clearly in the energy band diagram shown in Scheme 1. Photoactive SbNRs act as cosensitizers, undergo electron–hole separation under irradiance, and provide additional charge carriers, thereby contributing to improving the photocurrent and the PCE of the solar cell. Time-resolved fluorescence decay curves of SbNRs and SiNWs and SbNRs@SiNWs were measured at λ_em_ = 450 nm, and they are shown in Figure 3d. The decay profiles were fitted into a double exponential function, and the parameters are summarized in Table S2. The average lifetimes obtained for SiNWs, SbNRs, and SbNRs@SiNWs are 3.2, 3.2, and 2.5 ns, respectively. The lifetime in pristine SbNRs or SiNWs represents the residence time in their respective CBs prior to radiative recombination.

The shorter lifetime of SbNRs@SiNWs is due to fast electron injection from SbNRs to SiNWs (if SiNWs are not present, then the lifetime is longer because band edge recombination is a slower process compared to electron transfer between the CBs). The fast transfer is due to the thermodynamically favorable positions of the CBs of SiNWs (−4.1 eV) and SbNRs (−3.44 eV). Under irradiance, the excited electrons are injected from the CB of SbNRs to the CB of SiNWs, and these electrons are transferred to the external circuit when used in a solar cell, thus accounting for the high efficiency of this cell. Therefore, the excited electron lifetime is shorter for SbNRs@SiNWs. Simultaneously, SbNRs show the p-type conduction mechanism, which was confirmed by a Mott–Schottky analysis. The 1/*C*^2^ versus potential plot shows a linear behavior with a negative slope. The data is shown in Figure S7.

The VBs of SiNWs (−5.2 eV) and SbNRs (−5.09 eV) are well aligned for the hole transfer. Under irradiance, holes are also easily transported from SiNWs via SbNRs to the electrolyte. Thus, SbNRs increase the charge separation in the photoanode.

The dc electrical conductivities (σ) of SbNRs were measured under dark and light in the FTO/SbNRs/SS configurations (cartoon in Figure S8a), and the corresponding *I*–*V* plots are shown in Figure S8a. In this setup, a parafilm spacer of thickness “l” separated the two current collectors, and SbNRs were filled into a cavity of area “a” created at the center to prevent the shorting of the cell. White light illumination (100 mW cm^–2^) was done from the FTO side. While dark and light currents were not significantly different over the −1 to +0.5 V potential window, at ∼+0.5 V, a steep increase in current was registered only under illumination. Using linear fits over this region, the conductivities of SbNRs were estimated using Ohm’s law, σ = (Δ*I*/Δ*V*) × l/a, and the values were 2 and 0.2 μS cm^–1^, respectively. Charge separation under irradiance coupled with light-stimulated fast charge propagation possibly causes the sharp enhancement in current, thus imparting photoconductivity to SbNRs. This property is useful, for it can accelerate charge transport and transfer to SiNWs during solar cell operation. Conduction in dark in SbNRs is explained here. Sb is a metalloid comprising fused and ruffled, six-membered rings. The nearest and next-nearest neighbors form an irregular octahedral complex, with the 3-Sb atoms in each double layer relatively closer than the 3-Sb atoms in the adjacent one. The interlayer weak bonding allows electron transport between the layers, which is significantly amplified under irradiance. In the past, Sb-embedded carbon nanorods (CNRs) in a perovskite solar cell exhibited faster electron transport compared to sole CNRs.^36^ Sb incorporation made the work function shallower and also offered additional light scattering in Sb-CNR, thus resulting in increased PCE.^36^

To independently quantify the ability of SbNRs to serve as photosensitizers, *J*–*V* characteristics were measured for a film of SbNRs deposited over FTO (as the working electrode), and Pt rod as the CE, in dark and under 1 sun illumination in the presence of I^–^/I_3_^–^ liquid electrolyte (cartoon in Figure S8b represents the cell configuration). The *J*–*V* plots in Figure S8b show that the photocurrent density increased by 19 times from 0.02 to ∼0.4 mA cm^–2^ and the photovoltage of the cell improved by 1.3 times from 0.30 to 0.38 V, ongoing from the performance in dark to that in light. This result proves that upon illumination semiconducting SbNRs undergo electron–hole separation, and the photoexcited electrons are transferred to FTO, and via the external circuit, they reach the Pt electrode, where I_3_^–^ ions in the electrolyte reduce to I^–^ ions at the Pt/electrolyte interface. Thereafter, I^–^ ions diffuse to the SbNRs/FTO electrode, are oxidized, and regenerate the SbNRs, thus completing the circuit and the cell functions as a solar cell. All of these processes are triggered by illumination and occur spontaneously.

### Cu_2_O NCs: Structure and Nature

**Figure 4:**
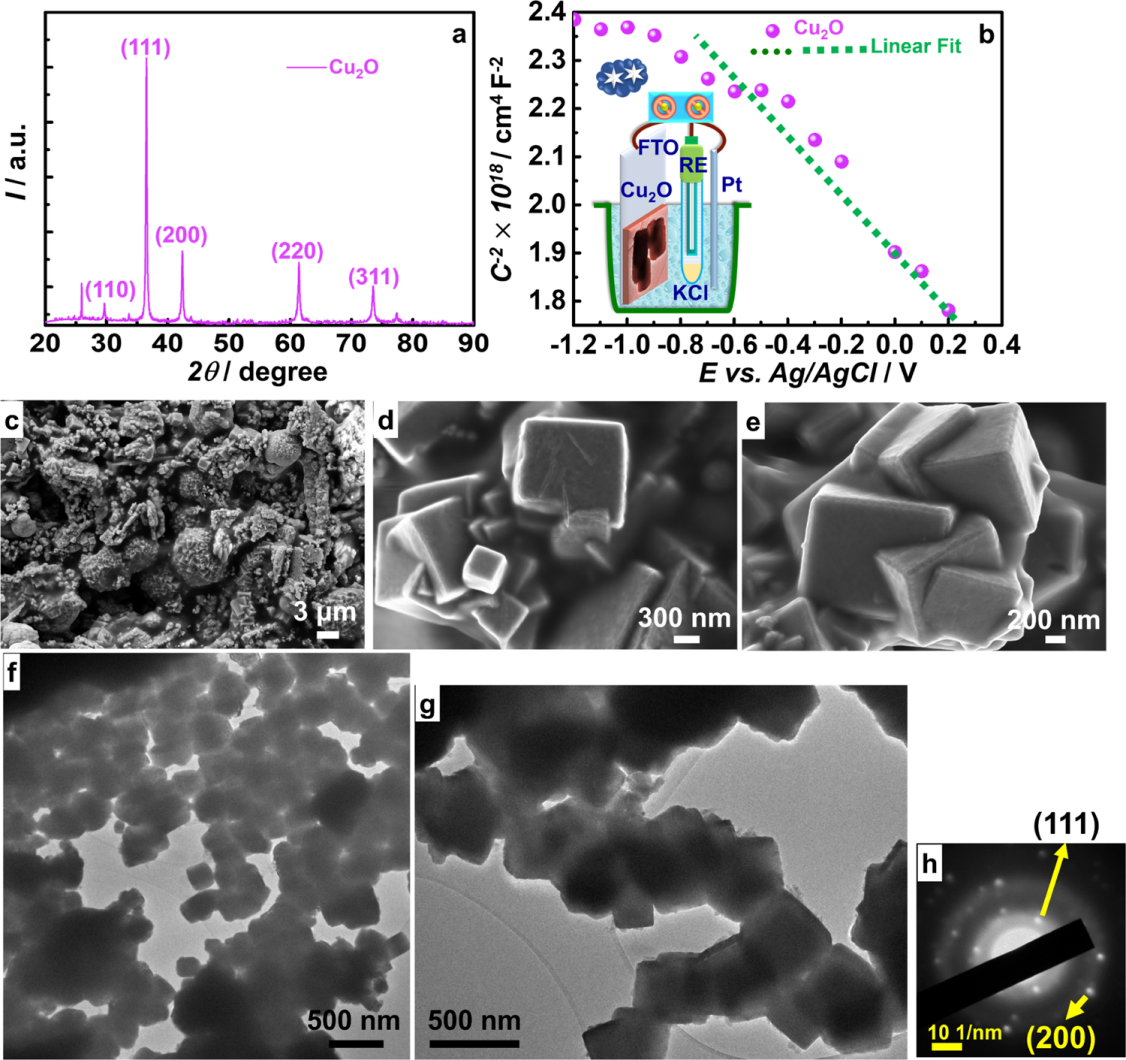
(a) XRD pattern and (b) Mott–Schottky plot of Cu_2_O NCs; the inset of (b) is a cartoon of the cell used. (c–e) FE-SEM images, (f,g) TEM images, and (h) SAED pattern of Cu_2_O NCs.

The crystal structure of Cu_2_O was determined from the XRD pattern (Figure 4a), and the diffractogram shows distinct peaks at 2Θ = 29.78, 36.56, 42.36, 61.44, and 73.53°, which are assigned to the (110), (111), (200), (220), and (311) planes, respectively. These are indexed to the cubic phase having the lattice constant *a* = 4.3 Å (JCPDS 05-0667). In Cu_2_O, each O-atom is surrounded by 4 Cu-atoms, and each Cu-atom is bonded by 2 O-atoms and it is preferentially oriented along the (111) facet. This facet is positively charged, for 2 Cu-atoms possess a dangling bond, which is at 90°. It is also known that the Cu_2_O crystal with a purely (111) facet is photocatalytically more active than other facets,^37^ which is again beneficial for the present cell.

Further, the p-type nature of Cu_2_O was confirmed by the Mott–Schottky plot (Figure 4b) recorded for a Cu_2_O/FTO film in a 0.1 M KCl electrolyte with Pt as the CE and Ag/AgCl/KCl as the reference electrode (RE) in dark (cartoon in Figure 4b depicts the cell used). The Mott–Schottky equation is given as follows
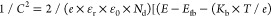
2

In the above equation, *C* is the capacitance, ε_r_ is the dielectric constant (taken as 7.6 from an earlier report),^38^ ε_0_ is the permittivity of free space, *E*_fb_ is the flat band potential, and *k*_b_ is Boltzmann’s constant. The slope is negative, implying a p-type conduction, and the magnitude is given by 2/(*e* × ε_r_ × ε_0_ × *N*_d_). From the slope, the hole density (*N*_d_) is estimated at 4.35 × 10^13^ cm^–3^. *E*_fb_ corresponds to the intercept and is found to be 0.25 V versus Ag/AgCl/KCl, which is close to the CB of Cu_2_O.

FE-SEM images of Cu_2_O are shown in Figure 4c–e, and it is made up of cubic shapes lumped together. The cubes have well-defined plane-facets with sharp edges and they are fused into each other to form a network of large 3D-irregular-shaped particles on micron-scale. These images clearly evidence the highly crystalline nature of Cu_2_O. TEM images (Figure 4f,g) affirm that at the nanoscale, Cu_2_O is indeed made up of interconnected densely packed cube-like shapes with dark contrast (suggesting compactly packed Cu_2_O crystallites therein) and the dimensions are in the range of 100–400 nm. The SAED pattern (Figure 4h) shows bright spots distributed randomly over hazy rings, and the former are indexed to the (111) and (200) planes of the cubic phase.

### Role of Cu_2_O NCs in the Electrolyte

**Figure 5:**
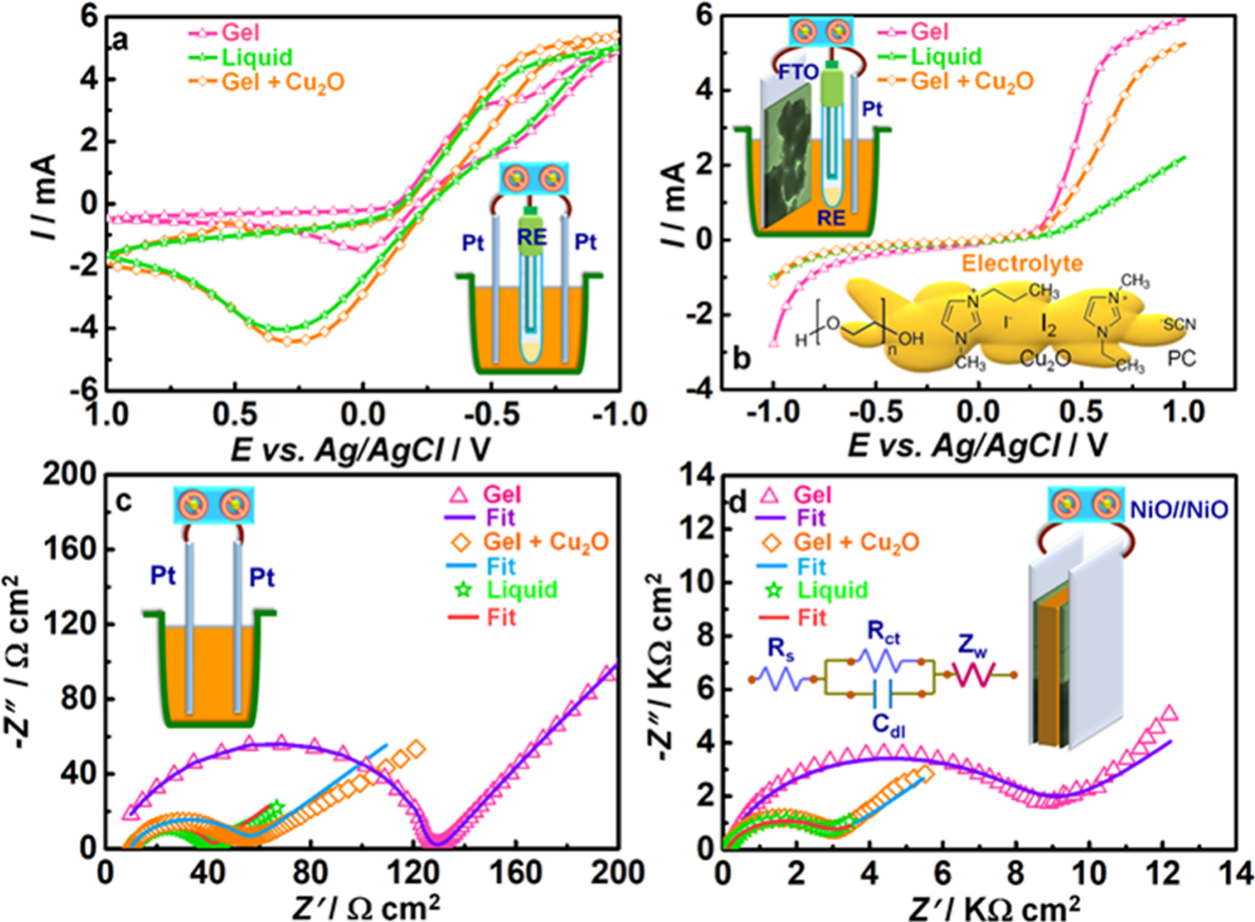
For liquid (I_2_ + I^–^ + PMII + EMISCN + PC), gel (I_2_ + I^–^ + PMII + EMISCN + PEO + PC), and Cu_2_O-gel (I_2_ + I^–^ + PMII + EMISCN + PEO + Cu_2_O NCs + PC) electrolytes: (a) CV and (b) LSV plots recorded at 10 and 100 mV s^–1^, respectively, Nyquist plots recorded in (c) Pt//Pt and (d) NiO//NiO configurations at *V*_OC_ and *V*_ac_ = 20 mV over 1 MHz to 0.01 Hz. Insets of panels (a–d) are cartoons representing the cell used in that measurement. The inset of panel (b) shows the Cu_2_O-gel components. The inset of panel (d) is the equivalent circuit.

The electrochemical behaviors of the liquid (I_2_ + I^–^ + EMISCN + PMII + PC), gel (I_2_ + I^–^ + EMISCN + PMII + PEO + PC), and Cu_2_O–gel (I_2_ + I^–^ + EMISCN + PMII + PEO + Cu_2_O NCs + PC) electrolytes were compared, and the cartoons in the insets of Figure 5 depict the cell configurations employed in each measurement. CV plots were recorded in a 3-electrode cell with two Pt rods as the WE and CE and a Ag/AgCl/KCl as the RE immersed in the said electrolyte (Figure 5a). The CV plots show a broad reduction peak in the cathodic branch corresponding to the following reaction: 1/2I_3_^–^ + e^–^ = 3/2I^–^, which were observed at 0.54, 0.24, and 0.46 V versus SHE, respectively. Interestingly, the Cu_2_O-gel shows reduction of I_3_^–^ ions at a higher positive potential relative to the pristine gel, which proves that the I_3_^–^ species can be more spontaneously reduced to I^–^ in the presence of Cu_2_O. Furthermore, the peak current densities are comparable for Cu_2_O-gel and the liquid electrolytes and higher than that of the gel, thus indicating that the propensity of Cu_2_O-gel to electrocatalyze I_3_^–^ reduction is quite high. The standard reduction potential for I_3_^–^ to I^–^ reduction is 0.35 V versus SHE.^39^ The oxidation peak is only observed for the Cu_2_O-gel electrolyte at 0.50 V versus NHE. Iodine being a halogen atom is very reluctant to undergo oxidation, and it generally has a strong tendency to readily capture one electron to convert to I^–^, i.e., the overpotential for the reverse reaction, iodide conversion to iodine is very large. So, for this reason in most of the electrolytes, only the reduction peak is observed. In the case of the Cu_2_O-gel, it is apparent that Cu_2_O possibly alters the interfacial composition to make it more conducive for iodide oxidation.

LSV studies were performed with NiO as the WE, Pt as the CE, and Ag/AgCl/KCl as the RE in liquid, gel, and Cu_2_O-gel electrolytes (Figure 5b). The onset of reduction occurs at 0.54, 0.48, and 0.37 V versus SHE for the liquid, Cu_2_O-gel, and gel electrolytes, respectively, which matches the outcome of CV analysis. The overpotential of reduction for liquid, Cu_2_O-gel, and gel electrolytes is 0.19, 0.13, and 0.02 V, respectively. NiO provides a larger active area for interaction with the electrolyte due to its mesoporous structure. Also, it acts as a highly electrocatalytic material allowing fast electron injection into the electrolyte. It has also been widely used in dye-sensitized solar cells as a photocathode.^40^ Finally, with NiO, a greatly enhanced PCE is obtained, thus justifying its use as a CE.

Nyquist plots for symmetrical cell configurations with two Pt electrodes immersed in liquid, gel, and Cu_2_O-gel are shown in Figure 5c. The data were fitted into an [R(RC)W] circuit. The charge-transfer resistances (*R*_ct_) for the cells are ∼32, ∼118, and ∼47 Ω cm^2^, respectively. *R*_ct_ is the highest for the gel and lowest for the liquid electrolyte, and the Cu_2_O-gel gives a value close to that of the liquid. In the gel, the polymer, PEO, retards charge transport, which possibly affects both *R*_ct_ and also makes the I_3_^–^ reduction potential more negative, as seen earlier. However, when Cu_2_O NCs are dispersed in the gel, due to their intrinsic hole-conducting capability, they offset the effect of PEO, enhance charge transport, and also facilitate charge transfer at the Pt/electrolyte interface. The oxidation peak obtained for Cu_2_O versus Ag/AgCl is +1.1 V. Hence, the HOMO level is at −5.79 eV. The HOMO level of Cu_2_O is perfectly aligned with the HOMO level of NiO at −5.3 eV for electron conduction to take place. The electron from the VB of NiO gets quickly captured by the VB of p-type Cu_2_O, which helps in lowering the charge-transfer resistance at the counter electrode/electrolyte interface. This is the reason why the R_ct_ drops drastically upon Cu_2_O addition to the gel electrolyte.

Furthermore, their high degree of crystallinity also imparts additional mechanical strength to the gel, thus enabling the fabrication of quasi-solid-state solar cells. With NiO//NiO symmetric cells, the same performance was repeated albeit slightly higher values registered for the charge-transfer resistances. From Figure 5d, it can be gauged that the *R*_ct_ magnitudes are higher compared to Pt//Pt cells. Compared to Pt, the sheet resistance of p-type NiO is significantly larger than that of Pt, and this impacts the *R*_ct_ value. The fitted parameters for NiO//NiO and Pt//Pt cells with different electrolytes are furnished in Table S3.

### Properties of NiO

NiO/FTO was deployed as the CE in the SiNW-based solar cells. The NiO film was deposited over FTO in chronoamperometric mode, with Ag/AgCl/KCl as the RE and a Pt rod as the CE, by applying a constant dc potential for 10 min (cartoon in Figure S9a represents the cell used). The current versus time transient is shown in Figure S9a and corresponds to the following reactions^41^
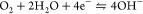
3
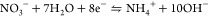
4
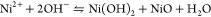
5

The initial spike in current is due to the reduction of the nitrate ions at the working electrode, which leads to the formation of OH^–^. The OH^–^ ions react with Ni^2+^ cations to produce Ni(OH)_2_. The formation of insoluble Ni(OH)_2_ at the working electrode leads to a lowering in current. After some time, the current saturates and then increases as the ion diffusion process predominates.

Current versus *t*^–1/2^ plot is provided as an inset. Using the Cottrell equation and from the linear fit shown in Figure S9a, the diffusion coefficient (*D*) for the migrating species was determined to be 7.99 × 10^–12^ cm^2^ s^–1^
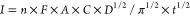
6

Here *F* is 96 500 C/mol, *A* is the active area, *C* is the concentration of the analyte, and *n* is the number of electrons involved in the redox process.

The UV–visible absorption spectrum of NiO (Figure S9b) shows a strong λ_max_ at 312 nm, and the optical band gap calculated from the equation *E*_g_ = 1240/λ for NiO is 3.6 eV, and it is an indirect band-gap semiconductor.^42^ The XRD pattern of NiO (Figure S9c) exhibits a set of broad peaks, indicative of a semicrystalline structure positioned at 2Θ = 36.99, 43.68, 63.17, 74.69, and 79.76°, which are attributed to the (111), (200), (220), (311), and (222) planes of the fcc crystal structure of NiO and the intensity variation also matches well with the standard diffraction pattern (JCPDS 04-0835). The Mott–Schottky analysis of NiO (Figure S9d) shows that it is a p-type semiconductor with a hole density of 9.40 × 10^13^/cm^3^ calculated from eq 5, where ε_r_ of NiO was taken as 12 from a previous report.^43^

FE-SEM images (Figure S10a–c) of NiO clearly bring out the mesoporous structure, as the oxide appears to be composed of irregularly shaped particles separated by pores to 10–30 nm in dimensions. TEM images show that the particles are composed of cubic or cuboidal shapes, with size in the range of 30–90 nm (Figure S10d). An interfringe distance of 0.20 nm is observed in a high-resolution image (Figure S10e), and it aligns with the (220) reflection of the fcc phase of NiO. The SAED pattern (Figure S10f) shows scattered spots, which are indexed to the (220), (311), and the (222) planes of the same structure. These results are in good agreement with the XRD data.

## Photovoltaic Performances of SiNW-Based Solar Cells

### Effect of the TMSPA Monolayer

The surface of SiNWs after etching undergoes partial oxidation, which leads to the formation of -OH functional groups on the nanowire surface. The -OH-functionalized SiNWs are highly vulnerable in liquid electrolyte. To prevent the degradation of such -OH functional groups via electrolyte corrosion and photocorrosion, it is very important to functionalize the -OH groups on the surface. N-[3-(Trimethoxysilyl)propyl]aniline or TMSPA coating on as-fabricated SiNWs leads to the formation of a covalently linked self-assembled monolayer over the SiNW surface through the O–Si bonds. The mechanism is shown in Scheme S2, and because the molecule is only a few nanometers in size, this monolayer is a few nanometers thick and is invisible to the naked eye.

XPS analysis of SbNRs@TMSPA@SiNWs was performed to clearly understand the coordination between TMSPA and SiNWs (Figure S6, Supporting Information). The full survey spectrum shows the presence of Si 2p, Sb 4s, N 1s, O 1s, and Sb 3d at 99, 148, 401, 530, and 539 eV, respectively. The core level of Sb 3d upon deconvolution shows the presence of Sb 3d_5/2_, Sb 3d_3/2_, and O 1s at 526, 535, and 529 eV. The obtained values are very similar to the previously reported values.^44^ The relative intensities of Sb 3d_5/2_ and Sb 3d_3/2_ are 33.27 and 18.09%, respectively. The core level spectrum of N1s show one asymmetric peak, and upon deconvolution, the peaks are assigned to the C–N and N–H bonds (arising specifically from the TMSPA) at 399 and 401 eV, respectively. The relative intensities of C–N and N–H bonds are 86.2 and 13.8%, respectively. The presence of N1s peaks in TMSPA@SbNRs@SiNWs even after the cleaning step confirms the bonding between TMSPA and SiNWs.

**Figure 6:**
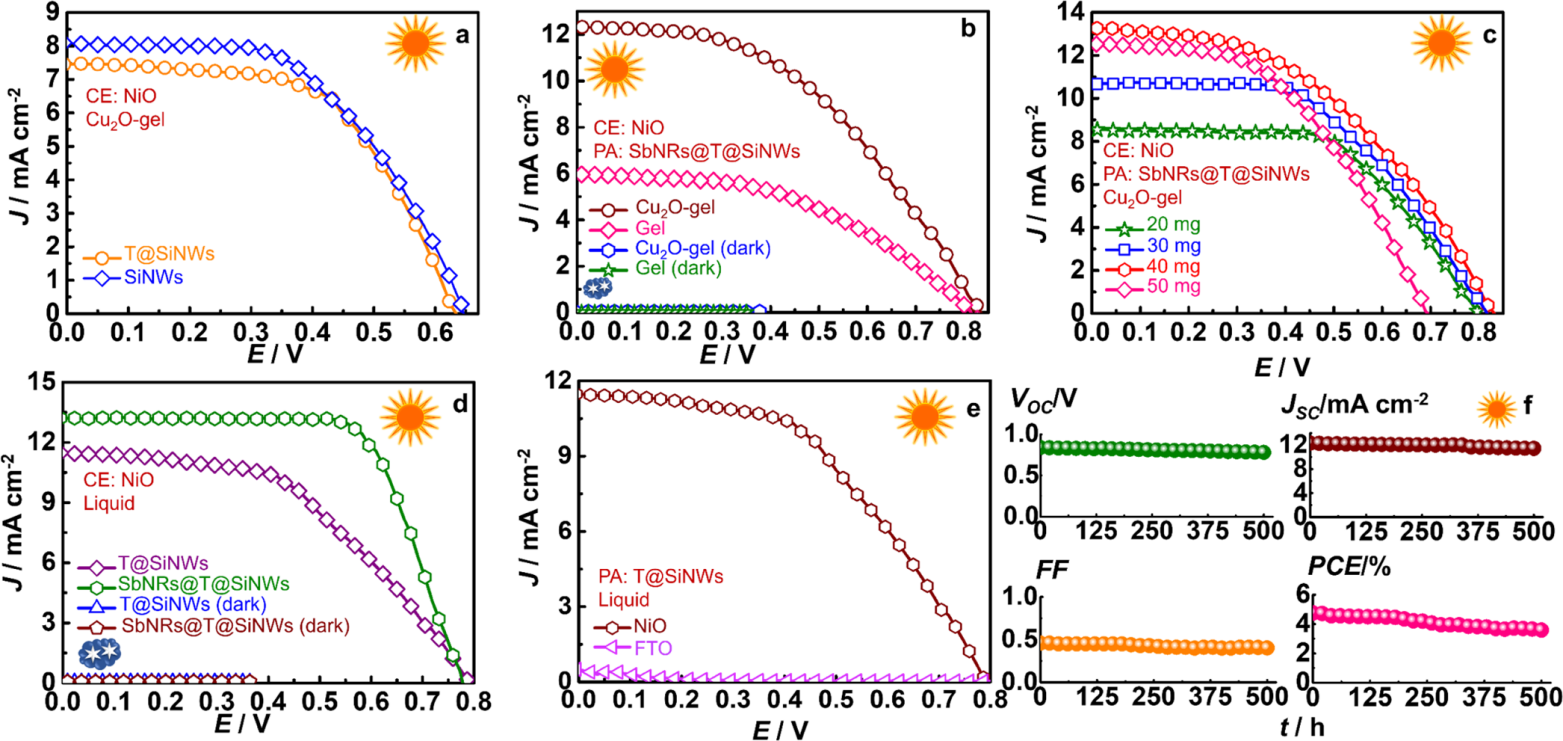
*J*–*V* plots of SiNW-based solar cells with different photoanodes (PAs), CEs, and electrolytes: (a) SiNWs with and without the TMSPA (T) monolayer, (b) with and without Cu_2_O NCs in the gel, (c) optimization of the Cu_2_O NC content in the gel, (d) with and without SbNRs in the liquid electrolyte, (e) with two CEs: NiO and FTO. (f) Variation in *V*_OC_, *J*_SC_, FF, and PCE of SbNRs@TMSPA@SiNWs/Cu_2_O-gel/NiO cell under intermittent illumination for 500 h.

The *J*–*V* characteristics of TMSPA@SiNWs/Cu_2_O-gel/NiO and SiNWs/Cu_2_O-gel/NiO solar cells, essentially with and without the TMSPA monolayer, are compared in Figure 6a. The process improves solar cell stability and operational lifetime, without compromising the PCE for they are 2.7 and 2.8%, thus unambiguously illustrating that it is a powerful yet simple technique to enhance cell stability, an issue of great concern, particularly with regard to commercial prospects. The solar cell parameters are provided in Table 1, and the five cell average data with standard deviation is given in Table S4. The obtained standard deviation value is close to the value obtained by Ahn et al.^45^ for their perovskite solar cells. It is observed that the variation in the values ongoing from one cell to another is not much; this is probably because here a liquid or a gel electrolyte is used as the HTL. With such an HTL, deep penetration into the photoanode and CE is ensured because they are porous electrodes. This results in superior interfacial properties compared to solid-state cells (like organic solar cells), and multiple cells can therefore show performance parameters that are close in values.

**Table 1:** Solar Cell Parameters of the Champion Cells, and the Average Value for Each Parameter in Parenthesis

cell architecture	*V*_OC_ (mV)	*J*_SC_(mA cm^–2^)	FF	PCE (%)
Effect of NiO on the Solar Cell Performance				
TMSPA@SiNWs/liquid/NiO@FTO	791	11.5	0.49	4.4
TMSPA@SiNWs/liquid/FTO	890	0.4	0.08	0.03
Effect of Cu_2_O on the Solar Cell Performance				
SbNRs@TMSPA@SiNWs/gel/NiO@FTO	811	6.0	0.45	2.2
SbNRs@TMSPA@SiNWs/Cu_2_O-gel/NiO@FTO	840	12.3	0.46	4.7
Effect of TMSPA on the Solar Cell Performance				
TMSPA@SiNWs/Cu_2_O-gel/NiO@FTO	629	7.5	0.58	2.7
SiNWs/Cu_2_O-gel/NiO@FTO	651	8.1	0.53	2.8
Effect of SbNRs on the Solar Cell Performance				
SbNRs@TMSPA@SiNWs/liquid/NiO@FTO	771	12.9	0.72	7.2
TMSPA@SiNWs/liquid/NiO@FTO	791	11.5	0.49	4.4
Effect of Gel on the Solar Cell Performance				
SbNRs@TMSPA@SiNWs/liquid/NiO@FTO	771	12.9	0.72	7.2
SbNRs@TMSPA@SiNWs/gel/NiO@FTO	811	6.0	0.45	2.2

### Effect of Cu_2_O NCs in the Gel

To study the effect of Cu_2_O NCs in controlling the solar cell performance, *J*–*V* characteristics of two SbNRs@TMSPA@SiNWs//NiO cells encompassing (i) pristine gel (without Cu_2_O) and (ii) the Cu_2_O-gel were compared (Figure 6b). Best cell parameters of *V*_OC_ = 811 mV, *J*_SC_ = 6 mA cm^–2^, FF = 0.45, and PCE = 2.2% with pristine gel and *V*_OC_ = 840 mV, *J*_SC_ = 12.3 mA cm^–2^, FF = 0.46, and PCE = 4.7% with Cu_2_O-gel were obtained. The PCE with Cu_2_O-gel is more than twice that without Cu_2_O. To better understand the role of Cu_2_O NCs in bringing about cell performance improvement, the content of Cu_2_O in the gel was first optimized by comparing SbNRs@TMSPA@SiNWs/x mg Cu_2_O-gel/NiO cells (*x* = 20, 30, 40, and 50) in Figure 6c and Table S5. It is observed that at 40 mg Cu_2_O content, the PCE is the highest. The Cu_2_O-gel has the Cu_2_O NCs homogeneously dispersed throughout the gel, and when the gel comes in contact with the photoanode, the Cu_2_O NCs also come in contact with the SiNWs or the SbNRs. At this point, the p-type conducting behavior of Cu_2_O NCs coupled with the suitably positioned (or more positive) Fermi level of Cu_2_O relative to the VBs of SiNWs or SbNRs (see Scheme 1) allows rapid hole extraction from the photoanode. The holes are then immediately transferred to the I^–^ ions, which undergo oxidation and diffuse to the CE. At Cu_2_O content >40 mg in the cell, the PCE drops, possibly due to aggregation of Cu_2_O NCs, which introduces inhomogeneity in the gel composition, and renders the oxide to be less effective as a hole scavenger. Nonuniform aggregation of Cu_2_O NCs at high contents retards hole transfer and transport and lowers the PCE.

### Effect of SbNRs in a Liquid Electrolyte Cell

To study the role of SbNRs in ameliorating the solar cell performance, *J*–*V* characteristics of SbNRs@TMSPA@SiNWs/liquid/NiO and TMSPA@SiNWs/liquid/NiO were compared (Figure 6d). The cell devoid of SbNRs showed *V*_OC_ = 791 mV, *J*_SC_ = 11.5 mA cm^–2^, FF = 0.49, and PCE = 4.4% and the one with SbNRs showed *V*_OC_ = 771 mV, *J*_SC_ = 12.9 mA cm^–2^, FF = 0.72, and PCE = 7.2%. With the liquid electrolyte and with SbNRs, not only a high PCE is obtained, but a high FF, in excess of 70% is achieved, which is a remarkable achievement. In the past for SiNW-based LJSCs, such a high FF has rarely been observed;^1,3,6^ FFs typically of about 40–55% have been obtained in the best SiNW cells. *J*_SC_ increases by ∼12% for SbNR-decorated solar cells. This increase in current is due to the p-type hole transport property, which lowers the electron–hole recombination at the photoanode/electrolyte interface. Thus, SbNRs assist in improving charge separation, and this is reflected in the FF increment, which increases quite significantly by ∼47%. A very high *J*_SC_ with a low FF generally does not represent a good solar cell. Since here a good balance between both *J*_SC_ and FF is achieved with the addition of SbNRs, this cell has strong potential for practical applications.

SbNRs are semiconducting, and as shown earlier, they are highly fluorescent with strong emission in the visible region and are therefore capable of undergoing charge separation under irradiance. The excited charge carriers are then rapidly transferred to the CB of SiNWs, permitted thermodynamically by the energy level offsets of SiNWs and SbNRs. SiNWs then transmit these electrons to the external circuit. Simultaneously, the holes produced in SiNWs transfer to SbNRs and then to I^–^ in the electrolyte, maximizing charge separation and suppressing electron–hole recombination, thereby maximizing the PCE, which is 7.2%. This leads to efficient light-generated carrier separation and a smooth single pathway for hole transportation via favorably aligned energy levels. These factors also reduce the recombination at the photoanode/electrolyte interface and improve solar cell efficiency compared to the pristine SiNW photoanode-based solar cell, wherein recombination can occur very easily. The liquid nature of the electrolyte is also beneficial, for PCE was only 2.2% with gel, and it is 7.2% with the liquid, having the same photoanode of SbNRs@TMPSA@SiNWs and a CE of NiO/FTO. While the gel enables the fabrication of a quasi-solid-state cell with ease, which is also relatively easier to handle, but the liquid offers better permeation properties, it is able to easily infiltrate the cross sections of the photoanode (be it SiNWs or SbNRs@SiNWs) and the mesoporous NiO CE, thus maximizing the interaction between the VBs of the photoanode and the I^–^ ions. The deep penetration of the liquid electrolyte compared to that of gel allows for more charge separation improving the PCE.

Despite the high PCE, the liquid electrolyte poses several practical issues, which restricts its commercial applicability. Under illuminated conditions, the solvent from the liquid electrolyte can evaporate or leak from the edges of the device, thus lowering the PCE. In contrast, the gel electrolyte can be visualized as a liquid electrolyte immobilized in a solid phase (polymer). Therefore, it offers almost liquid-like conductivity and the polymer chains trap the solvent molecules and prevent their evaporation, thus imparting long-term device stability. The stability of the liquid electrolyte-based cell is recorded (cell efficiency drops from 7.17 to 1.76%) and is shown in Figure S11 in the Supporting Information. The solar cell parameters are compared in Table 1, and the five cell average data with standard deviation is shown in Table S4. A comparison with literature values of cells with SiNW-based photoanodes is provided in Table S6, and it shows that our value in terms of attaining a good trade-off between stability and efficiency is quite good.

### Effect of p-Type NiO

The effect of NiO as the CE is discerned by comparing the *J*–*V* characteristics of two cells with the same photoanode and the electrolyte but different CEs (NiO/FTO and FTO) (Figure 6e). The TMSPA@SiNWs/liquid/NiO/FTO cell showed *V*_OC_ = 791 mV, *J*_SC_ = 11.5 mA cm^–2^, FF = 0.49, and PCE = 4.4% and the TMSPA@SiNWs/liquid/FTO cell showed *V*_OC_ = 890 mV, *J*_SC_ = 0.4 mA cm^–2^, FF = 0.08, and PCE = 0.03%. The PCE increases by 147-times on replacing FTO with NiO/FTO. The reason for this huge jump in PCE is this: FTO is just a current collector and does not offer any electrocatalytic properties, whereas NiO is highly electrocatalytic and also furnishes a high reaction surface area by the virtue of its mesoporous morphology (as seen earlier in the FE-SEM images). Consequently, during solar cell operation, a very large number of I_3_^–^ species are adsorbed at the NiO/electrolyte interface and also undergo reduction very easily, for the VB of NiO is more negative relative to the work function of FTO. During the cell operation, the electrons from the photoanode reach the CE, and from FTO, they cascade to the VB of NiO, and from the VB, they are quickly picked up by the I_3_^–^ ions that are in direct contact with the NiO surface. FTO is a planar current collector and does not offer this benefit; as a consequence, the electron injection efficiency is poor at the FTO/electrolyte interface, and the PCE is very low. The optical transmittance of FTO is higher than that of NiO, as shown in Figure S12. The higher optical transmittance in the FTO electrode results in better light penetration, thus amounting to a better charge buildup in the SiNW photoanode. As a consequence, the TMSPA@SiNWs/liquid/FTO cell shows greater photovoltage. In comparison, the NiO CE shows an optimal balance between optical transmittance and electrocatalytic property, and this advantage trumps the above aspect of FTO, thus resulting in an overall higher PCE with NiO.

NiO/FTO absorbs some part of the incoming solar radiation. These losses in incoming light can be further adjusted by optimizing the thickness of the NiO layer, which will be attempted in future. A thin film of NiO can provide high light transmittance in visible and NIR regions. This can be achieved by lowering the electrodeposition time of the NiO film. Hammad et al. prepared NiO on the glass substrates/Si wafer by DC-sputtering method.^46^ They showed that the optical band gap of NiO films decreases as the film thickness increases; the thin film showed higher and broader UV–visible–NIR optical transmittance compared to the thick films. Chen et al. also reported the preparation of NiO films by DC magnetron sputtering.^47^ They showed that the films prepared at 100 W for 10 min showed the best optical transmittance (∼80%) over the visible–NIR region.

The solar cell parameters are compared in Table 1, and the five cell average data with standard deviation is shown in Table S4. Additionally, a *J*–*V* measurement was also performed with a Pt/FTO CE. SbNRs@TMSPA@SiNWs/Cu_2_O-gel/Pt@FTO cell and the *J*–*V* diagram are shown in Figure S13. The device showed *V*_OC_ = 870 mV, *J*_SC_ = 17.40 mA cm^–2^, FF = 0.63, and η = 9.40%. However, Pt is very expensive, and comparatively, NiO is very cheap.

### Effect of the Etching Time

The lengths and diameters of the SiNWs can be varied by varying the etching time of SiNW synthesis.^12−14^ Si wafers were etched for 5, 10, 15, and 20 min, and *J*–*V* parameters of the cell having configuration SbNRs@TMSPA@SiNWs (*x* min)/Cu_2_O-gel/NiO@FTO were measured. The *J*–*V* curves are shown in Figure S14 (Supporting Information), and the PV parameters are summarized in Table S7. It has been observed that the device efficiency increases from 3.05 to 4.7% upon increasing the etching time from 5 to 15 min. This could be due to the increase in the antireflective properties of the SiNWs upon an increase in wire length due to higher etching time. However, a further increase in etching time leads to the formation of ultralong nanowires, which get heavily bundled due to their long lengths. The bundling of nanowires causes poor electrolyte as well as light penetration into the electrode. This results in less charge separation and lower device efficiency and at 20 min etching time, the efficiency drops to 3.4%. A similar trend has been reported previously by many groups.^12−14^ The optimal average length of 3–7 μm is therefore achieved at an etching time of 15 min, which is the case here.

### Comparison of EQEs

The EQE spectra of SbNRs@TMSPA@SiNWs/Cu_2_O-gel/NiO@FTO and TMSPA@SiNWs/gel/NiO@FTO cells are now shown in Figure S15 (Supporting Information). The comparison shows that with SbNRs and Cu_2_O, a larger EQE is registered over the 400–1040 nm wavelength region. The EQE maxima with and without SbNRs and Cu_2_O are 38.7 and 9.7% at 880 nm, respectively.

### Stability Analysis

SiNW-based photoelectrochemical cells showed serious stability issues in the past. Lewis et al., in the year 2010, reported a SiNW-based solar cell where they used a viologen-based electrolyte, which showed a 3% efficiency but poor stability.^48^ In the past, the ferrocene/ferrocenium redox couple electrolyte dissolved in acetonitrile or methanol or water has been explored but it also suffered from poor stability.^49−53^ Several research groups reported the poor stability of SiNW-based photoelectrochemical cells in the aqueous HBr-Br_2_ electrolyte.^1,3^ The poor stability of the aqueous Br_2_/Br^–^ electrolyte-based device is attributed to the corrosive nature of HBr mineral acid and liquid bromine. SiNWs tend to rapidly dissolve in this corrosive medium, thus restricting the use of the solar cell for any practical application. Another point of concern with an aqueous electrolyte-based cell is that the SiNW photoanode gets partially oxidized to SiO_2_, which is highly resistive and causes the efficiency of the device to decline with time. Use of nonaqueous organic electrolytes based on solvents like propylene carbonate can solve the problem. In 2010, Shen et al. reported a photoelectrochemical cell where they used LiI and I_2_ dissolved in EMISCN and PMII ionic liquid electrolytes and the cell showed excellent stability under 1 sun^2^. The ionic liquids EMISCN and PMII show high thermal and chemical stabilities and they are also nonvolatile, which ensures that the composition of the electrolyte does not change with time. Aqueous electrolytes have a tendency to evaporate through the crevices causing the PCE to decline with time. In this work, we used a poly(ethylene oxide) or PEO-based gel electrolyte with LiI and I_2_ dissolved in EMISCN, PMII, and propylene carbonate with Cu_2_O NCs dispersed therein. The electrolyte synthesis is accomplished at low temperatures and it is a very stable electrolyte.

After 500 h of continuous illumination, the PCE of the SbNRs@TMSPA@SiNWs/Cu_2_O-gel/NiO@FTO cell dropped by ∼24.4% and the efficiency of the TMSPA@SiNWs/Cu_2_O-gel/NiO@FTO device dropped by ∼70.1%. Hence, the SbNRs not only improve the device efficiency but also passivate the SiNW surface by capturing the holes from SiNWs and protecting it from electrolyte corrosion. The stability data of the SbNRs@TMSPA@SiNWs/Cu_2_O-gel/NiO@FTO cell is presented in Table S8 of the Supporting Information.

The PCE of the TMSPA@SiNWs/Cu_2_O-gel/NiO@FTO device dropped by ∼70.1%, and the PCE of the SiNWs/Cu_2_O-gel/NiO@FTO device dropped by ∼96.8%. The higher drop in PCE of the device without TMSPA shows a greater drop in efficiency after 500 h. The lower drop in PCE in the TMSPA@SiNWs/Cu_2_O-gel/NiO@FTO cell is due to the self-assembled monolayer formation of TMSPA, which protects the SiNWs from photo-oxidation and electrolyte corrosion. The data is presented in Table S9 of the Supporting Information.

### Charge-Transfer/Transport Phenomena in Solar Cells

Nyquist plots of SbNRs@TMSPA@SiNWs/liquid/NiO under light and dark are shown in Figure S16a and are fitted into R(QR)(QR) and R(RQ)W circuits. Under illuminated conditions, the bulk resistance (*R*_b_) = 128 Ω cm^2^, *R*_ct_ at the CE/electrolyte interface = 185 Ω cm^2^, and recombination resistance (*R*_rec_) at the photoanode/electrolyte interface = 12.4 kΩ cm^2^. In dark, the *R*_b_ is unaltered, but *R*_ct_ = 5.4 kΩ cm^2^. *R*_ct_ increased by 29 times in dark compared to that in light. This increase in resistance is due to poor electron transport to counter electrode from photoanode under dark conditions. Nyquist plots of SbNRs@TMSPA@SiNWs/Cu_2_O-gel/NiO@FTO under light and dark are shown in Figure S16b, and the plots are fitted into an R(QR)(QR) circuit. Under illuminated conditions, the *R*_b_ = 59 Ω cm^2^, *R*_ct_ = 884 Ω cm^2^, and *R*_rec_ = 3650 Ω cm^2^ and in dark *R*_b_ = 56 Ω cm^2^, *R*_ct_ = 1125 Ω cm^2^, and *R*_rec_ = 5855 Ω cm^2^. The *R*_ct_ value increased 1.3 times, and *R*_rec_ value increased 1.6 times in dark compared to those in light. Under irradiance, the excited charged carriers recombine more compared to dark and as a result the cell showed higher recombination and charge-transfer resistance compared to light. The fitted parameters of all of the cells are summarized in Table S10. The Bode plots of SbNRs@TMSPA@SiNWs/liquid/NiO and SbNRs@TMSPA@SiNWs/ Cu_2_O-gel/NiO cells under dark and light are shown in Figure S16c. The recombination lifetime (τ) of the Cu_2_O-gel-based cell under dark and illuminated conditions is 11.3 and 0.6 ms, respectively, and the recombination lifetime with the liquid under illuminated condition is 24.1 ms.

## Conclusions

A quasi-solid-state solar cell with the following architecture, SbNRs@TMSPA@SiNWs/Cu_2_O-gel/NiO solar cell, was fabricated, and it delivered a PCE of 4.7%, superior by 177% compared to the cell without SbNRs or Cu_2_O NCs. Upon irradiance, SbNRs undergo electron–hole separation and inject excited electrons to SiNWs, while simultaneously accepting holes from them as well, thus minimizing recombination at the photoanode/electrolyte interface and improving photocurrents. Cu_2_O NCs, which are homogeneously dispersed in the gel matrix, also have a favorably poised Fermi level, allowing the unhindered extraction and transmission of holes from SbNRs or SiNWs to the I^–^ ions in the electrolyte. Electrocatalytic and mesoporous NiO as the CE affords substantial interaction with the I_3_^–^ ions and maximizes their reduction. The combined effect of these factors manifests the high PCE, and the TMSPA overlayer efficiently prevents the photocorrosion of SiNWs, enabling the cell to sustain 500 h of extended and intermittent illumination, with a 24.4% decline in its PCE. This study demonstrates the potential of using low-cost photoactive and/or semiconducting materials with favorably aligned energy levels for developing efficient solar cells, and these materials can easily conform to other solar cells as well.

## Supporting Information Available

Schematic of device fabrication, calculations of CB, VB positions from cyclic voltammetry study with band gaps, cross-sectional FE-SEM image of SiNWs, reflectance spectra of planar Si and SiNWs, Tauc plots, quantum yield calculation, time-resolved fluorescence lifetime values, SbNR Mott–Schottky, electrical conductivity and *J*–*V* measurement of SbNRs, Nyquist plot parameters, FE-SEM and TEM images of NiO, Mott–Schottky and XRD of NiO, schematic of TMSPA coating on SiNWs, XPS of photoanode, solar cell parameters with five cell averages, Cu_2_O optimization data, transmittance spectra of counter electrodes, stability plots of the liquid electrolyte-based cell, comparative table, cell average, *J*–*V* plots and Nyquist plots of solar cells, and stability data of various solar cells (PDF)
