# Supplementary material for: Effect of Cuprous Oxide Nanocubes and Antimony Nanorods on the Performance of Silicon Nanowire-Based Quasi-Solid-State Solar Cell
Source: ACS Omega. 2022 Dec 8;7(50):46311–24. doi: 10.1021/acsomega.2c04850 (PMC9773360; doi:10.1021/acsomega.2c04850)
Supplement: Supplementary file 1 — ao2c04850_si_001.pdf [file ao2c04850_si_001.pdf]

Supporting Information

Effect of Cuprous Oxide Nanocubes and Antimony Nanorods on the Performance of Silicon Nanowires Based Quasi Solid-State Solar Cell

Debanjan Maity,[a] Partha Ghosal,[b] Melepurath Deepa[a,*]

[a]Department of Chemistry, Indian Institute of Technology Hyderabad, Kandi, Sangareddy 502284, Telangana, India

[b]Defence Metallurgical Research Laboratory, Defence Research & Development Organisation (DRDO), Hyderabad, Telangana 500058, India

**Corresponding author. E-mail address: mdeepa@chy.iith.ac.in*

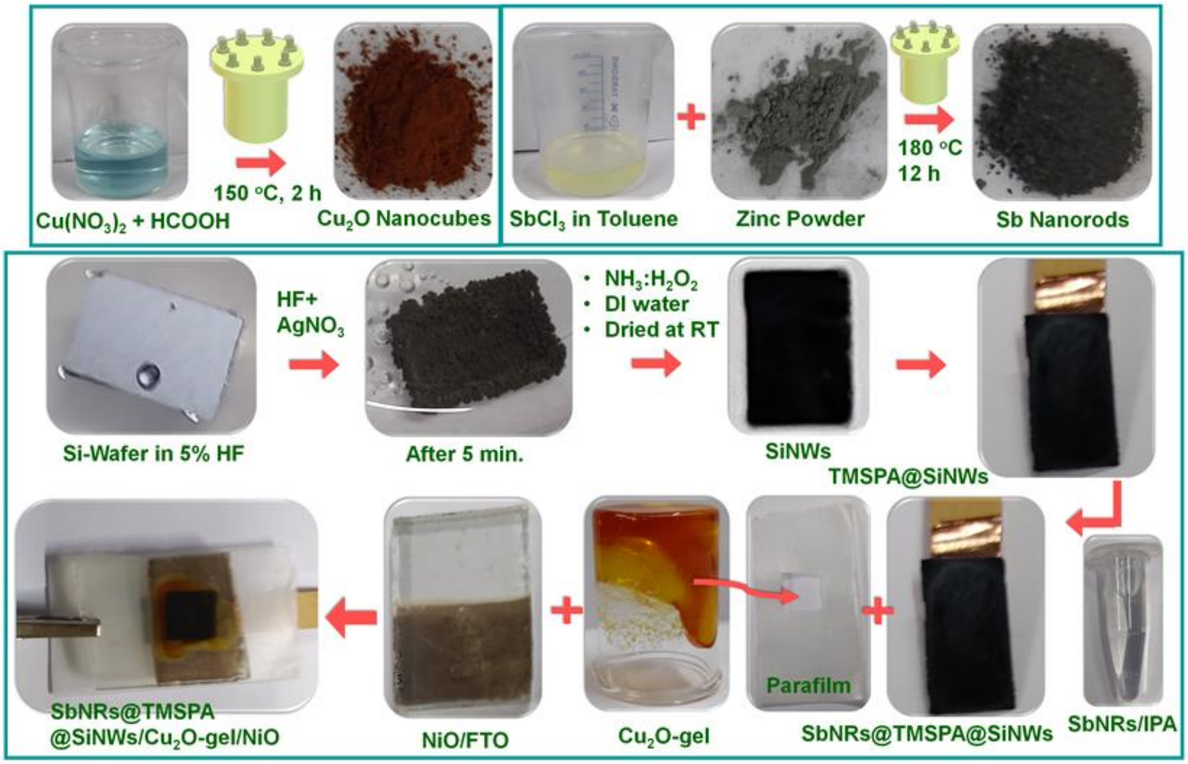

Scheme S1 Schematic presentation of the fabrication of the solar cell.

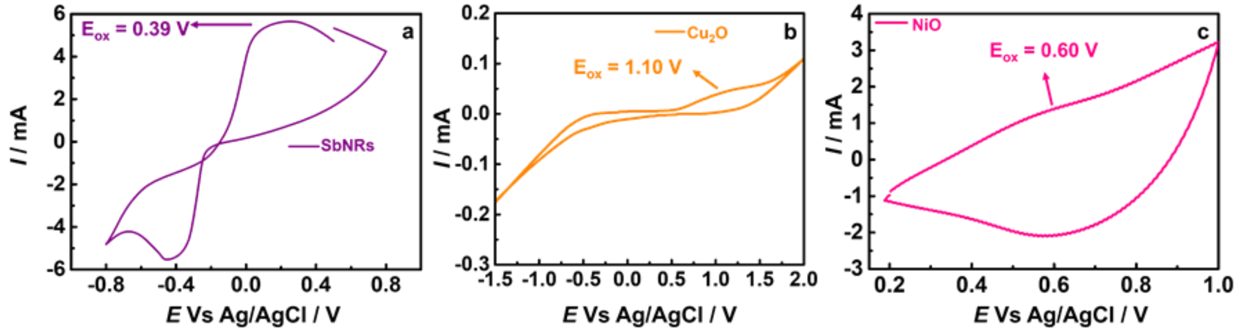

Figure S1 Cyclic voltammograms of (a) SbNRs/FTO, (b) Cu_2O/FTO and (c) NiO/FTO as the working electrodes, Pt as a counter electrode and a 0.1 M KCl aqueous solution as electrolyte and Ag/AgCl/KCl as the reference electrode, recorded at a scan rate of 10 mV/s.

The HOMO and LUMO levels of the materials used for fabrication are calculated using the cyclic voltammetry and absorbance spectra using the equation given below.

$$E_{red} = -4.5 \text{ eV } (= 0\text{V versus NHE}) - (\text{Red. Peak (V) vs. Ag/AgCl/KCl } +0.197) \quad (1)$$

$$E_{ox} = -4.5 \text{ eV } (= 0\text{V versus NHE}) - (\text{Ox. Peak (V) vs. Ag/AgCl/KCl } +0.197) \quad (2)$$

Table S1 Band positions of the photoanode components.

Material	Oxidation Peak / V vs. Ag/AgCl	Oxidation Peak / V vs. NHE	E_{red} (versus NHE) / eV = LUMO	Band Gap / eV	E_{ox} (versus NHE) / eV = HOMO
SbNRs	0.39	0.59	-3.44	1.65	-5.09
Cu_2O	1.10	1.30	-3.83	1.96	-5.79
NiO	0.60	0.80	-1.70	3.60	-5.30

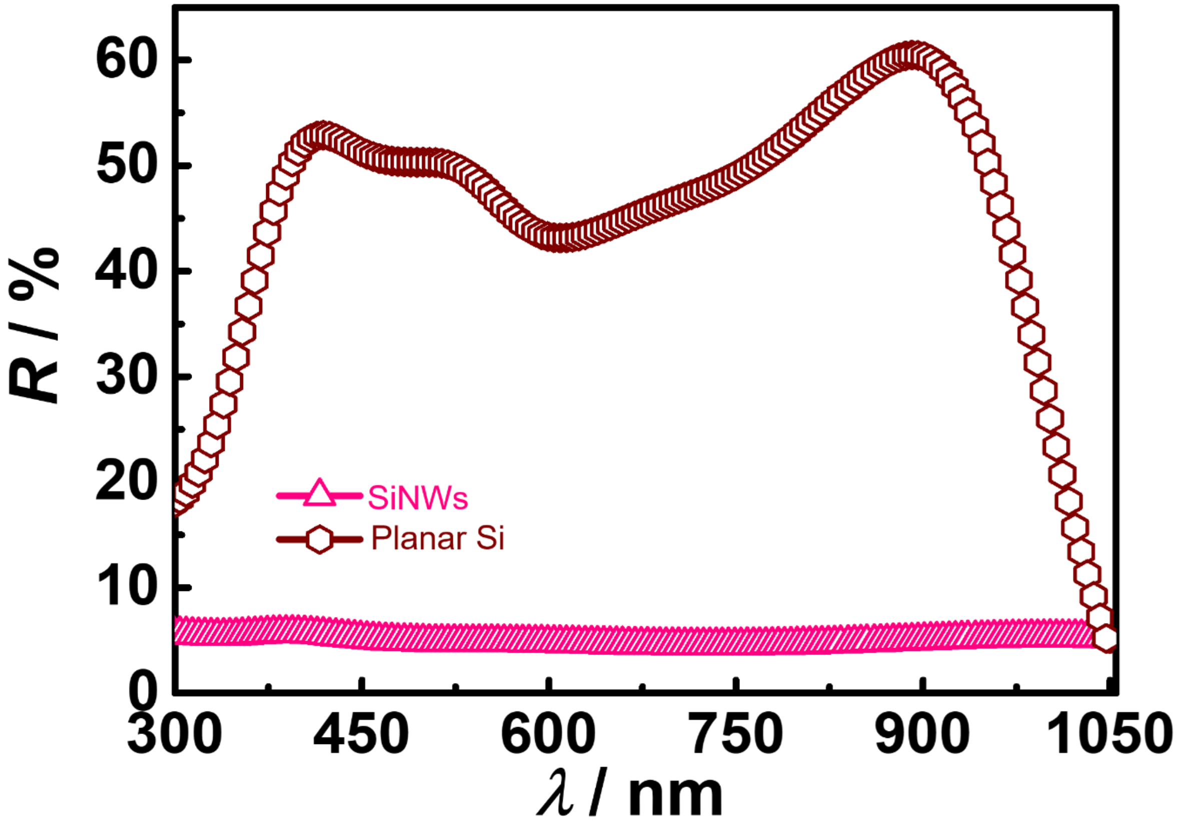

Figure S2: Reflectance spectra of SiNWs and planar Si.

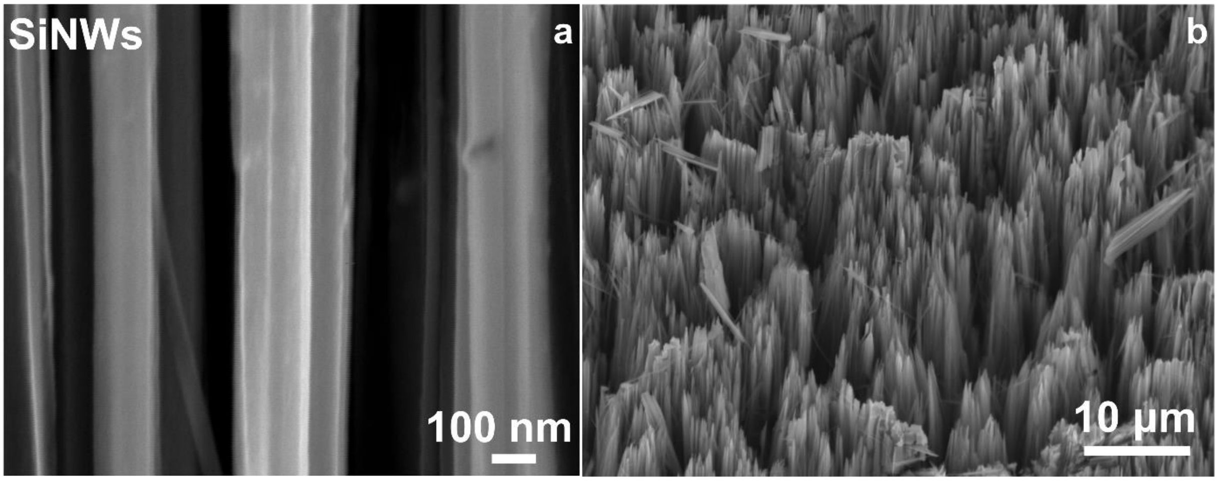

Figure S3 (a,b) Cross-sectional FE-SEM images of SiNWs.

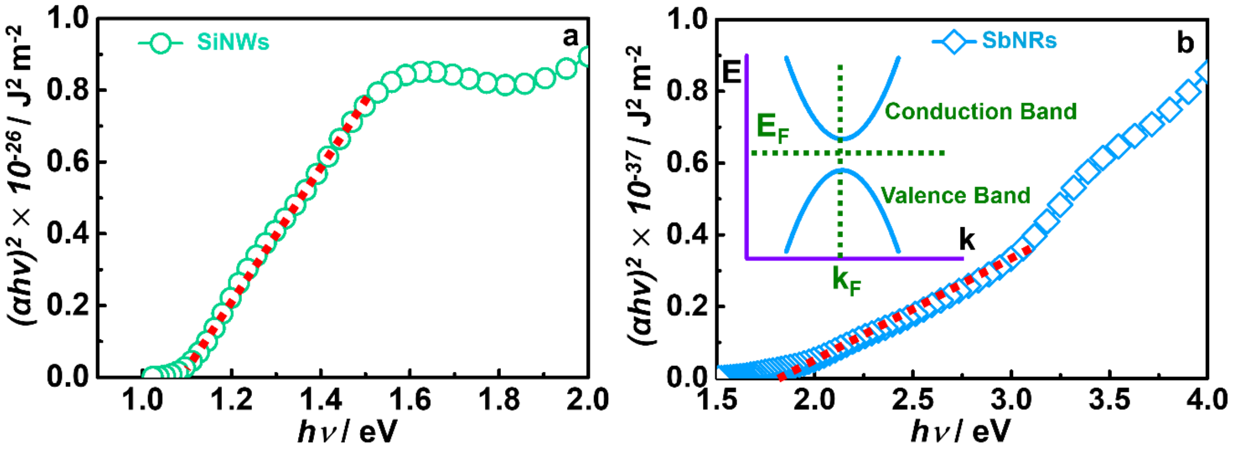

Figure S4 Tauc plots of (a) SiNWs and (b) SbNRs. Inset of (b) is for a direct E_g semiconductor.

The quantum yield of SbNRs was measured by using a solution of the Rhodamine 6G dye in ethanol as a reference. The absorbance and fluorescence spectra of SbNRs dispersion and Rhodamine 6G solution were measured at variable concentrations and Figure S1 shows the variation of integrated fluorescence intensity with absorbance.

The quantum yield of SbNRs is calculated using the following equation-

$$\varphi_X = \varphi_R \,(Grad_X/Grad_R)(\eta_X^2/\eta_R^2) \qquad (3)$$

where, φ_X is the quantum yield (unknown) of the material and φ_R is the quantum yield of the reference. The slope of the straight line obtained from the integrated fluorescence intensity versus absorption is “Grad”, and η is the refractive index of the solvents.

The known quantum yield of Rhodamine 6G is 0.95. The excitation wavelength used for the measurement is 370 nm. Hence from the experimental data the obtained value of quantum yield for SbNRs is 0.41.

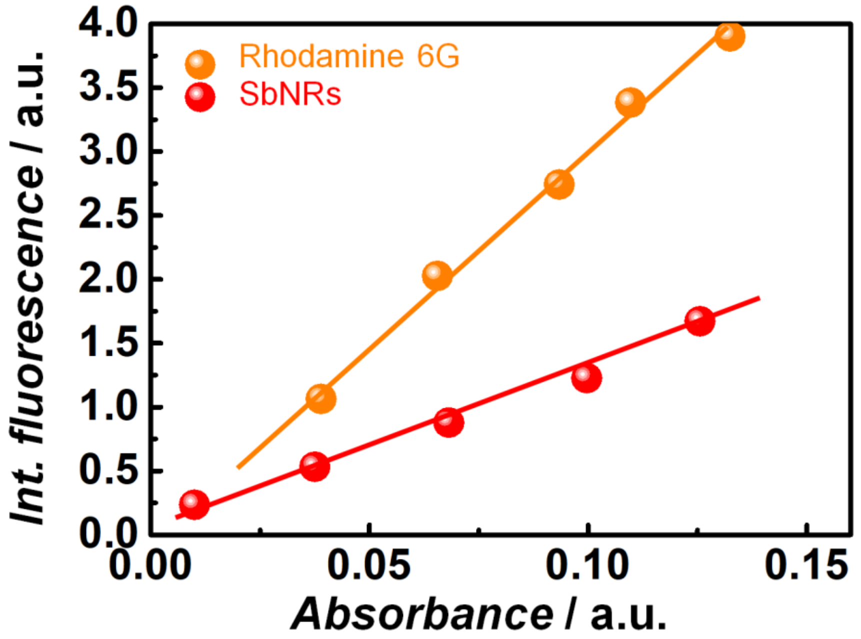

Figure S5 Integrated fluorescence intensity versus absorbance of SbNRs and Rhodamine 6G.

Table S2 Kinetic parameters of emission decay analysis of photosensitive materials and films obtained from double exponential fits.

Sample	χ^2	τ_1 (ns)	τ_2 (ns)	B_1	B_2	$<\tau>$	λ_{em} (nm)
SiNWs	1.33	1.4	12	97.6	2.4	3.2	450
SbNRs	1.25	1.0	7.4	93.2	6.8	3.2	450
SbNRs@SiNWs	1.20	0.8	5.5	92.1	7.9	2.5	450

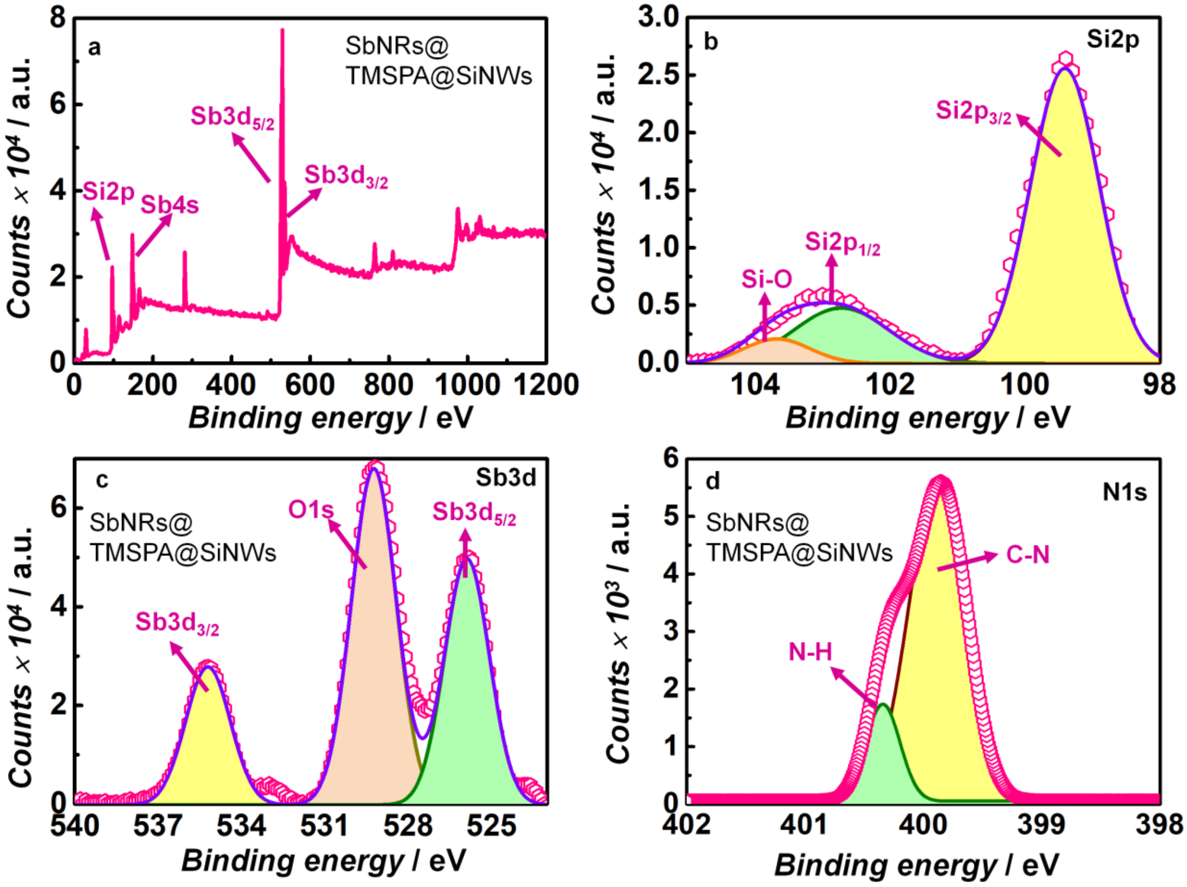

Figure S6 (a) survey spectrum and core level spectra of: (b) Si2p, (c) Sb3d and O1s and (d) N1s of SbNRs@TMSPA@SiNWs.

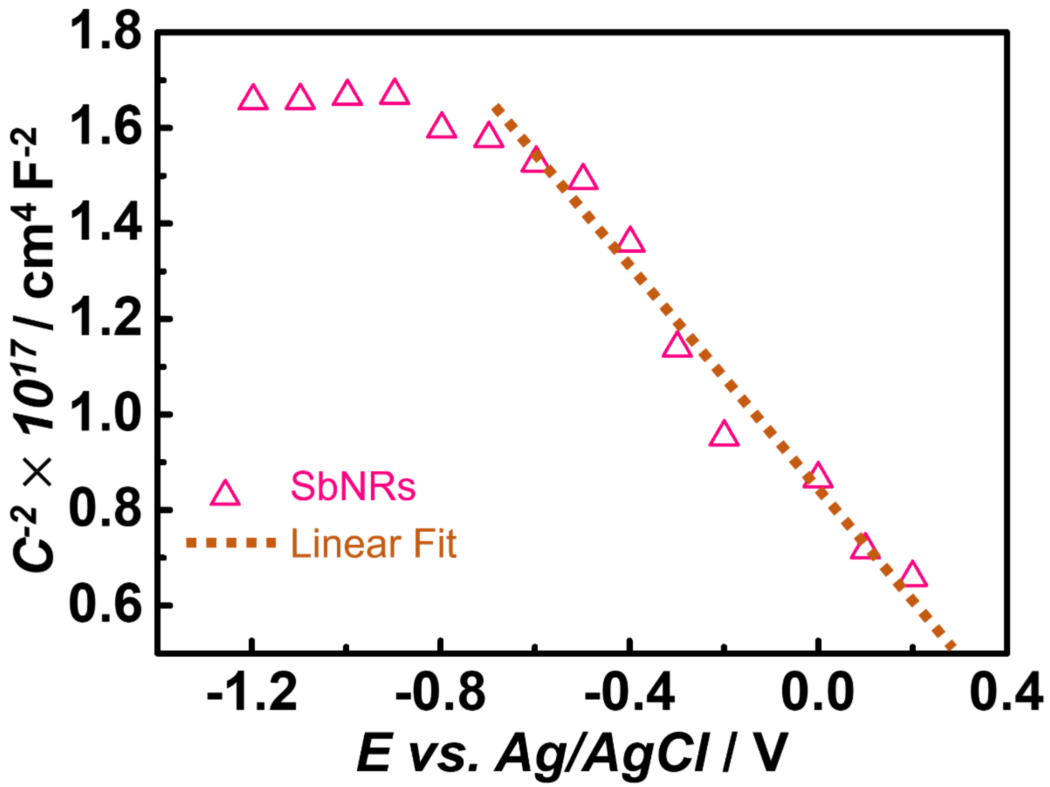

Figure S7 Mott-Schottky plot of SbNRs.

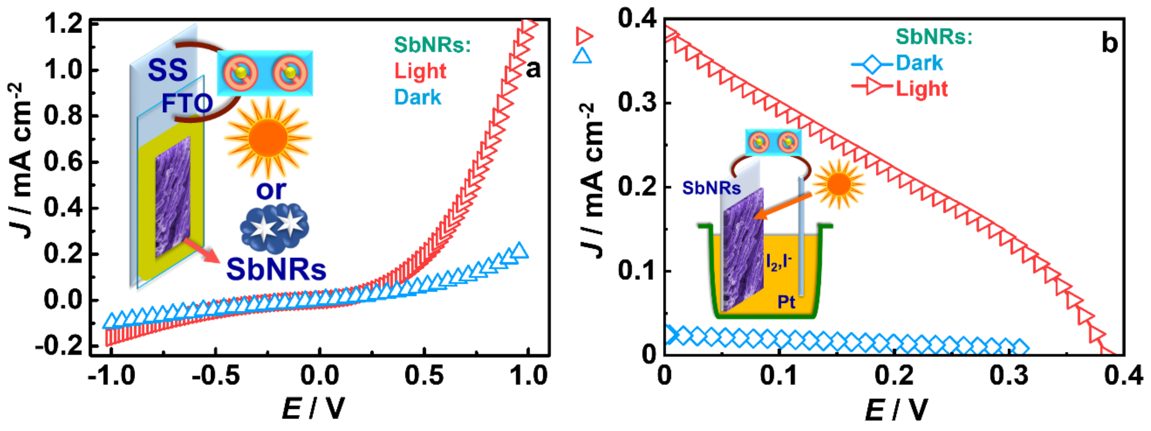

Figure S8 (a) I-V characteristics of SbNRs solid in a cell configuration shown in the inset cartoon. (b) J-V characteristics of SbNRs@FTO/liquid/Pt (inset cartoon shows the cell configuration used) under AM 1.5G, 1 sun illumination and in dark.

Table S3 Fitted parameters obtained from Nyquist plots for symmetric cells of the CEs.

Cells	R_b (Ω cm^2)	R_{ct} (Ω cm^2)	Y_0 (μΩ^{-1})	N	χ^2
Pt/liquid/Pt	9	32	93.6	0.8	9.01×10^{-2}
Pt/gel/Pt	10	118	31.1	0.8	1.84×10^{-4}
Pt/Cu_2O-gel/Pt	10	47	42.3	0.8	5.95×10^{-4}
NiO/liquid/NiO	116	2714	23.9	0.8	3.60×10^{-3}
NiO/gel/NiO	118	8344	15.1	0.8	1.01×10^{-3}
NiO/Cu_2O-gel/NiO	119	2807	24.8	0.8	1.30×10^{-3}

Liquid = I_2 + I^- + EMISCN + PMII + PC, Gel = I_2 + I^- + EMISCN + PMII + PEO + PC, Cu_2O-gel = I_2 + I^- + EMISCN + PMII + PEO + Cu_2O NCs + PC.

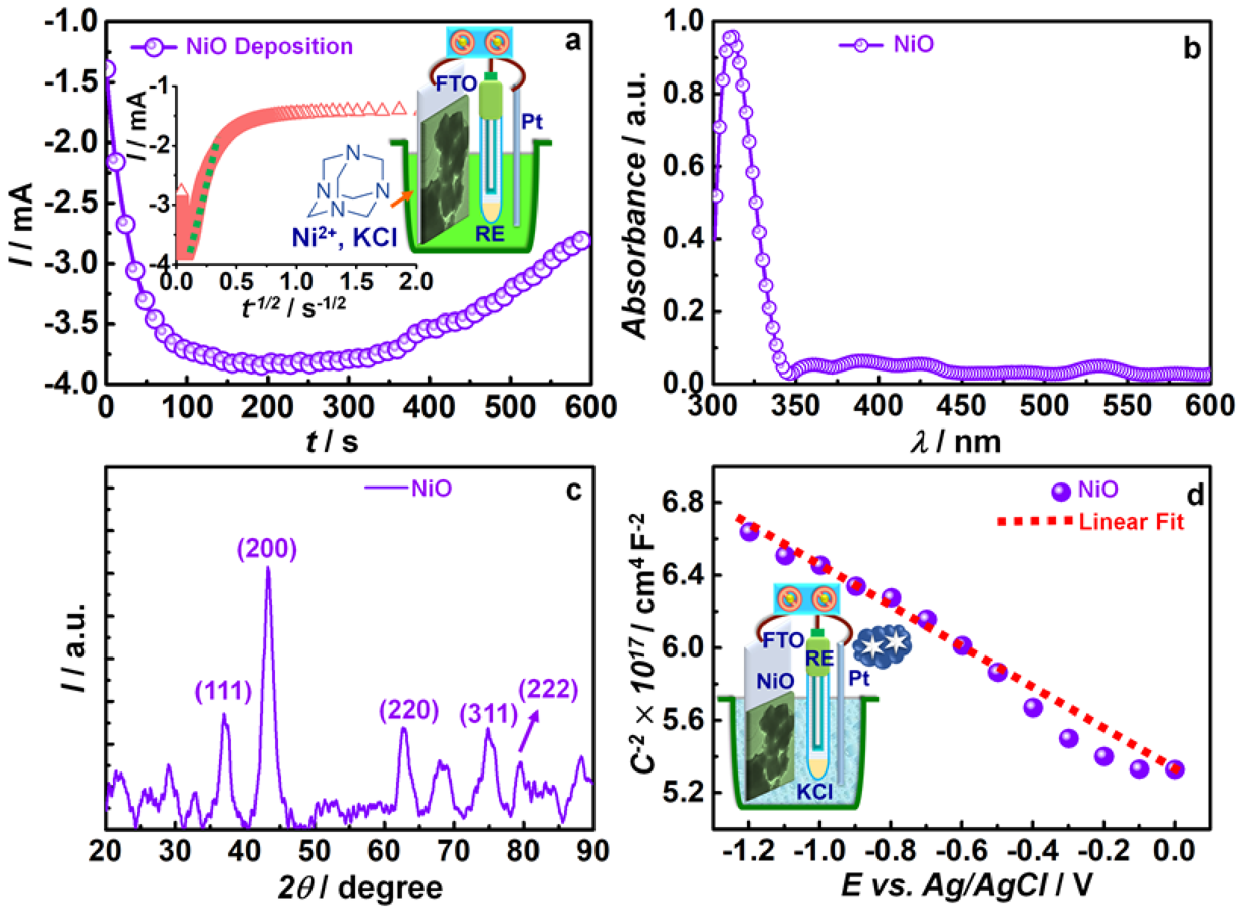

Figure S9(a) I versus t transient for NiO deposition, insets are a cartoon showing the cell used, and the corresponding I versus $t^{-1/2}$ plot. (b) Absorption spectrum and (c) XRD pattern of NiO. (d) Mott-Schottky plot of a NiO/FTO film; inset is a cartoon showing the cell used for the measurement.

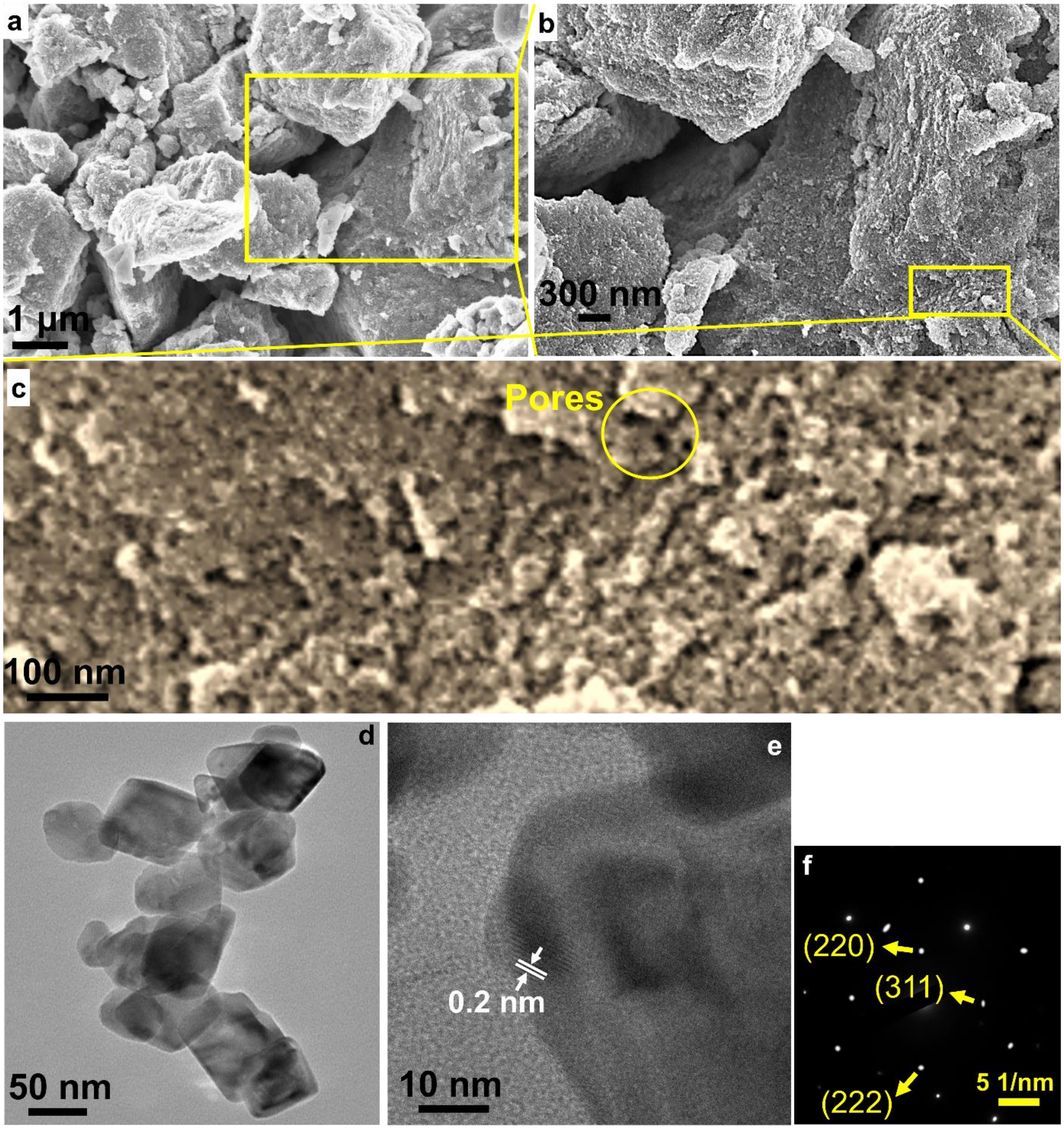

Figure S10 (a-c) FE-SEM images, (d) TEM image, (e) lattice scale image and (f) SAED pattern of NiO.

Scheme S2 Monolayer formation via covalent bonding with SiNWs by TMSPA.

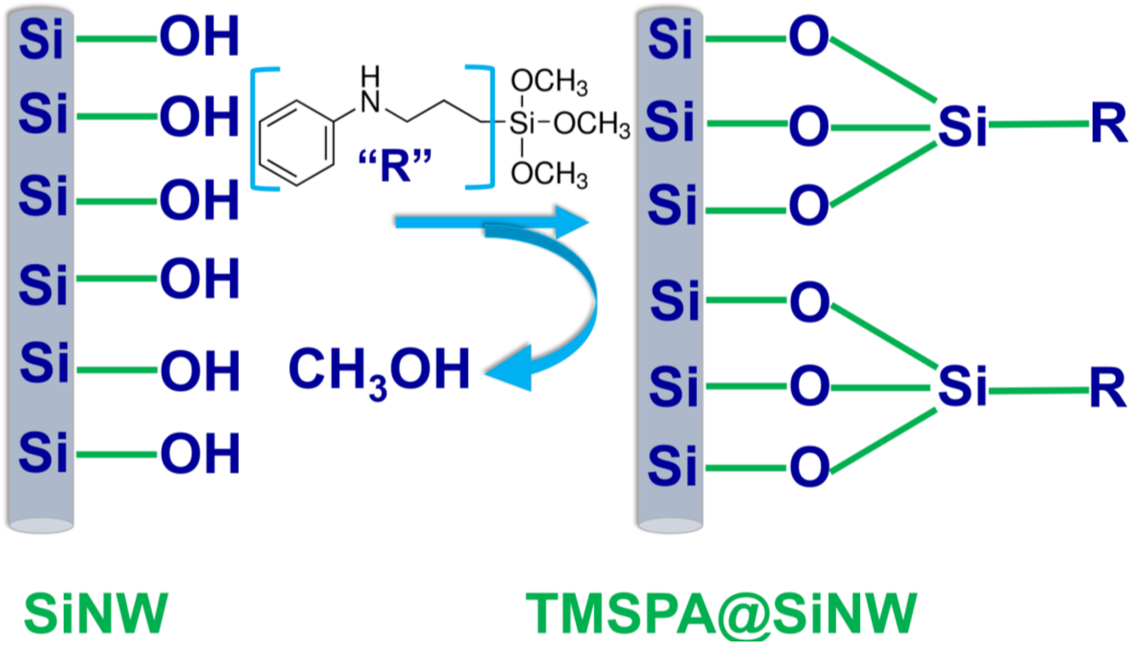

Table S4 Average values of solar cells parameters for different solar cells under AM 1.5G, 1 sun illumination.

Cells	V_{OC} (mV)	J_{SC} (mAcm^{-2})	FF	PCE (%)
TMSPA@SiNWs/I^-, I_3^- ionic liquid/NiO@FTO				
Cell 1	791	11.5	0.48	4.4
Cell 2	788	11.4	0.48	4.3
Cell 3	787	11.4	0.48	4.3
Cell 4	785	11.3	0.47	4.2
Cell 5	783	11.3	0.47	4.1
Average	787	11.4	0.48	4.3
Standard Deviation	2.72	0.08	0.01	0.11
TMSPA@SiNWs/I^-, I_3^- ionic liquid/FTO				
Cell 1	891	0.4	0.07	0.02
Cell 2	887	0.4	0.07	0.02
Cell 3	886	0.4	0.08	0.03
Cell 4	886	0.4	0.08	0.03
Cell 5	883	0.4	0.09	0.03
Average	887	0.4	0.08	0.03
Standard Deviation	2.61	0.01	0.008	0.01
TMSPA@SiNWs/I^-, I_3^- ionic liquid gel+Cu_2O/NiO@FTO				
Cell 1	631	7.5	0.58	2.7
Cell 2	627	7.5	0.58	2.7
Cell 3	626	7.5	0.58	2.7
Cell 4	624	7.5	0.57	2.7
Cell 5	620	7.5	0.57	2.6
Average	626	7.5	0.57	2.7
Standard Deviation	3.63	0.01	0.008	0.04
SiNWs/I^-, I_3^- ionic liquid gel/NiO@FTO				
Cell 1	759	3.3	0.69	1.7
Cell 2	757	3.3	0.66	1.6
Cell 3	755	3.3	0.65	1.6
Cell 4	756	3.2	0.64	1.6
Cell 5	754	3.2	0.60	1.5
Average	756	3.3	0.65	1.6
Standard Deviation	1.73	0.06	0.03	0.06
SiNWs/I^-, I_3^- ionic liquid gel+Cu_2O/NiO@FTO				
Cell 1	649	8.1	0.53	2.8
Cell 2	647	8.1	0.52	2.7
Cell 3	643	8.0	0.51	2.6

Cell 4	639	7.9	0.52	2.6
Cell 5	639	7.9	0.49	2.5
Average	643	8.0	0.51	2.6
Standard Deviation	4.09	0.08	0.04	0.11
SbNRs@TMSPA@SiNWs/I^-, I_3^- ionic liquid/NiO@FTO				
Cell 1	771	12.9	0.72	7.2
Cell 2	764	12.8	0.73	7.1
Cell 3	759	12.7	0.73	7.1
Cell 4	756	12.6	0.73	7.0
Cell 5	753	12.6	0.74	7.0
Average	761	12.7	0.73	7.1
Standard Deviation	6.36	0.12	0.02	0.08
SbNRs@TMSPA@SiNWs/I^-, I_3^- ionic liquid gel /NiO@FTO				
Cell 1	812	6	0.46	2.2
Cell 2	809	5.6	0.47	2.1
Cell 3	807	5.6	0.48	2.2
Cell 4	806	5.4	0.46	2.0
Cell 5	802	5.4	0.46	2.0
Average	807	5.6	0.47	2.1
Standard Deviation	3.32	0.22	0.03	0.09
SbNRs@TMSPA@SiNWs/I^-, I_3^- ionic liquid gel + Cu_2O /NiO@FTO				
Cell 1	840	12.3	0.46	4.8
Cell 2	836	12.3	0.46	4.7
Cell 3	835	12.2	0.45	4.6
Cell 4	833	12.0	0.44	4.4
Cell 5	832	11.9	0.44	4.4
Average	835	12.1	0.45	4.6
Standard Deviation	3.31	0.17	0.03	0.16

Table S5 Solar cell parameters of SbNRs@TMSPA@SiNWs/Cu_2O-gel/NiO@FTO cell at variable amounts of Cu_2O.

Cu_2O amount (mg)	V_{OC} (mV)	J_{SC} ($mAcm^{-2}$)	FF	η_{best} (%)
20	792	8.5	0.58	3.9
30	816	10.7	0.51	4.4
40	836	13.3	0.43	4.8
50	690	12.5	0.48	4.1

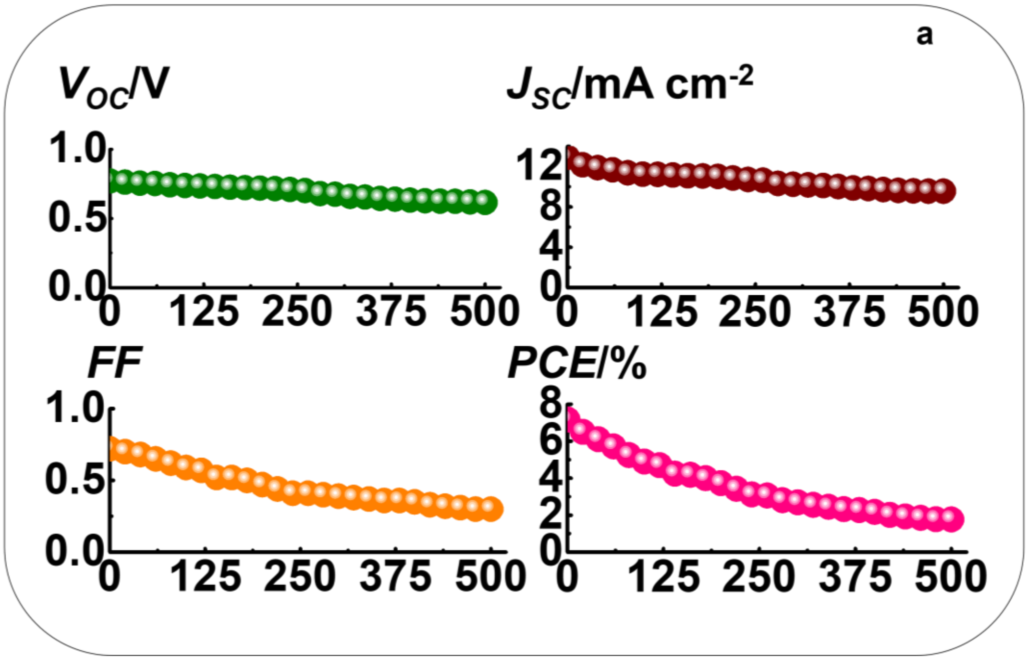

Figure S11 Variation in V_{OC}, J_{SC}, FF and PCE of SbNRs@TMSPA@SiNWs/liquid/NiO cell under intermittent illumination for 500 h.

Table S6 Comparison table of SiNWs based solar cell parameters reported in literature.

Solar Cell	V_{OC} (mV)	J_{SC} (mAcm^{-2})	FF	PCE(%)	Ref
PA: PtNPs@SiNW<br>CE: Pt mesh<br>Electrolyte: 0.05 M Br_2 in 8.6 M HBr (aq.)	580	17.2	0.61	6.1	[1]
PA: SiNW-CH3(Pt)<br>CE: Pt coated ITO<br>Electrolyte: 0.05 M I_2 & 0.1 M LiI in a mixed IL (EMISCN:PMII = 7:13, (v/v))	322	33.7	0.40	6	[2]
PA: PtNPs@C@SiNW<br>CE: Pt Mesh<br>Electrolyte: 0.05 M Br_2 in 8.6 M HBr (aq.)	530	36.9	0.55	10.9	[3]
PA: SeNPs@SiNWs	790	17.1	0.52	7	[4]

CE: C-fabric<br>Electrolyte: 0.05 M Br_2 in 8.6 M HBr (aq.)					
PA: C@TeNRs@SiNWs<br>CE: C-fabric<br>Electrolyte: 0.05 M Br_2 in 8.6 M HBr (aq.)	890	23.3	0.56	11.6	[5]
PA: SiNWs<br>CE: DWNT<br>Electrolyte: 3% Br_2 in 40% HBr	500	10.5	0.25	1.29	[6]
PA: SiNWs<br>CE: Pt mesh<br>Electrolyte:3% Br_2 in 40% HBr	750	0.9	0.45	0.29	[11]
Front contact: Cu<br>Absorber layer: SiNWs (Wire length: 450 nm)<br>Hole transporting layer: PEDOT<br>Rear contact: Al	460	26.7	0.54	6.9	[12]
Front contact: Ag<br>Absorber layer: SiNWs<br>Hole transporting layer: PEDOT<br>Rear contact: Al	465	30.6	0.65	9.3	[13]
Front contact: ITO<br>Absorber layer: SiNWs (Wire length: 370 nm)<br>Hole transporting layer: PEDOT<br>Rear contact: Ti/Ag	532	24.2	0.65	8.4	[14]
Front contact: ITO<br>Hole transporting layer: V_2O_5<br>Absorber layer: SiNWs<br>Electron transport layer: TiO_2<br>Rear Contact: Al	490	35.7	0.73	12.7	[21]
Front contact: Ag grid<br>Hole transporting layer: PEDOT: PSS<br>Absorber layer: SiNWs-DADS<br>Rear Contact: Al	488	28.8	0.49	7.02	[22]
PA: N-GQDs@PCDTBT@SiNWs<br>CE:C fabric<br>Electrolyte: 0.05 M Br_2 in 8.6 M HBr (aq.)	880	24.9	0.60	13.2	[23]
PA: ZnTPP@CdTe@SiNWs	790	19.5	0.59	9.1	[24]

CE: PEDOT-N(CF3SO$_3$)$_2$<br>Electrolyte: I^-, I_3^- gel					
PA: IL-GNP@SiNW<br>CE: WO_3<br>Electrolyte: I^-, I_3^- gel	768	18.4	0.56	7.9	[25]
PA: Single crystal Silicon matt-etched.<br>CE: Gold coated ITO (thin layer configuration)<br>Electrolyte:0.15-0.2 M 1,1'-dmf/ 0.15-0.2 M1,1'-dmtfb /1 M $LiClO_4$ –CH_3OH	600	34	0.69	14.0	[53]

ITO = Indium Tin Oxide, DWNT = Double Walled Carbon Nanotube, PEDOT = Poly(3,4-ethylenedioxythiophene), DADS = Diallyl disulfide, PCDTBT = poly[N-9′-heptadecanyl-2,7-carbazole-alt-5,5-(4′,7′-di-2-thienyl-2′,1′,3′-benzothiadiazole)], ZnTPP = 5,10,15,20-Tetraphenyl-21H,23H-porphine zinc, GNP = Graphene Nanoparticles, dmf = dimethyl ferrocene, dmftb = dimethyl ferrocene tetrafluoroborate.

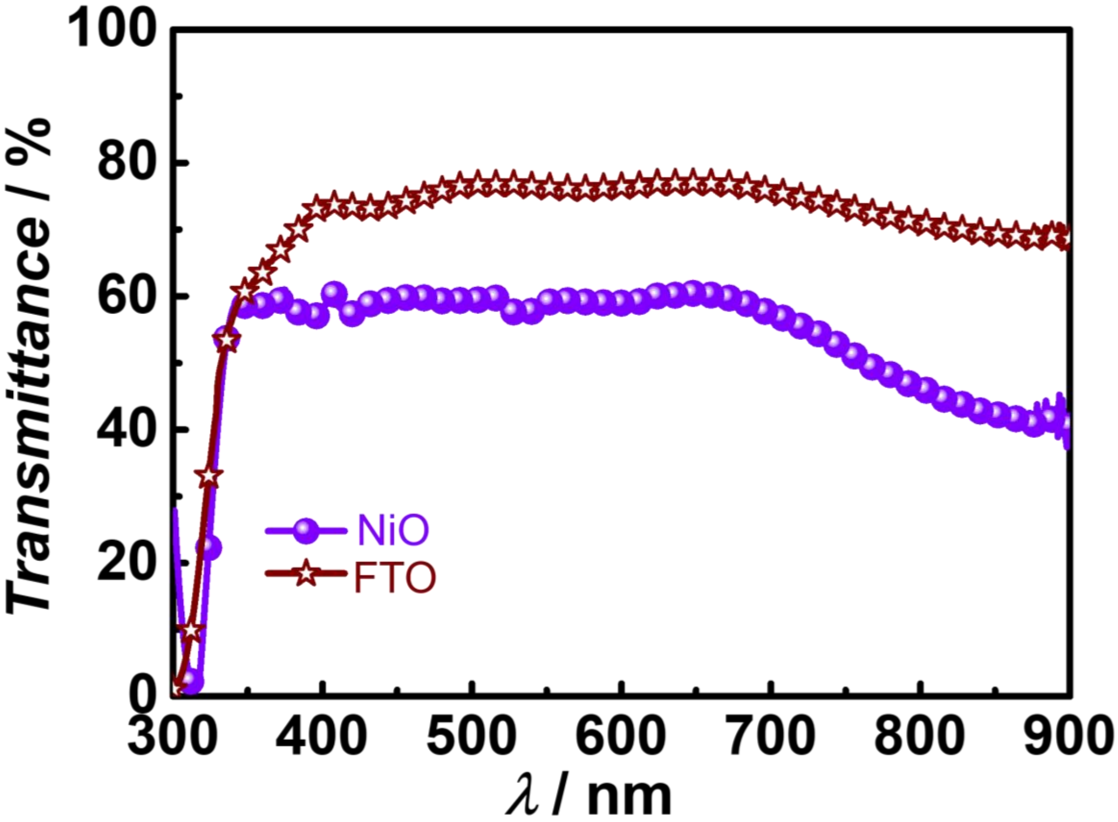

Figure S12 Transmittance spectra of NiO@FTO and pristine FTO electrodes.

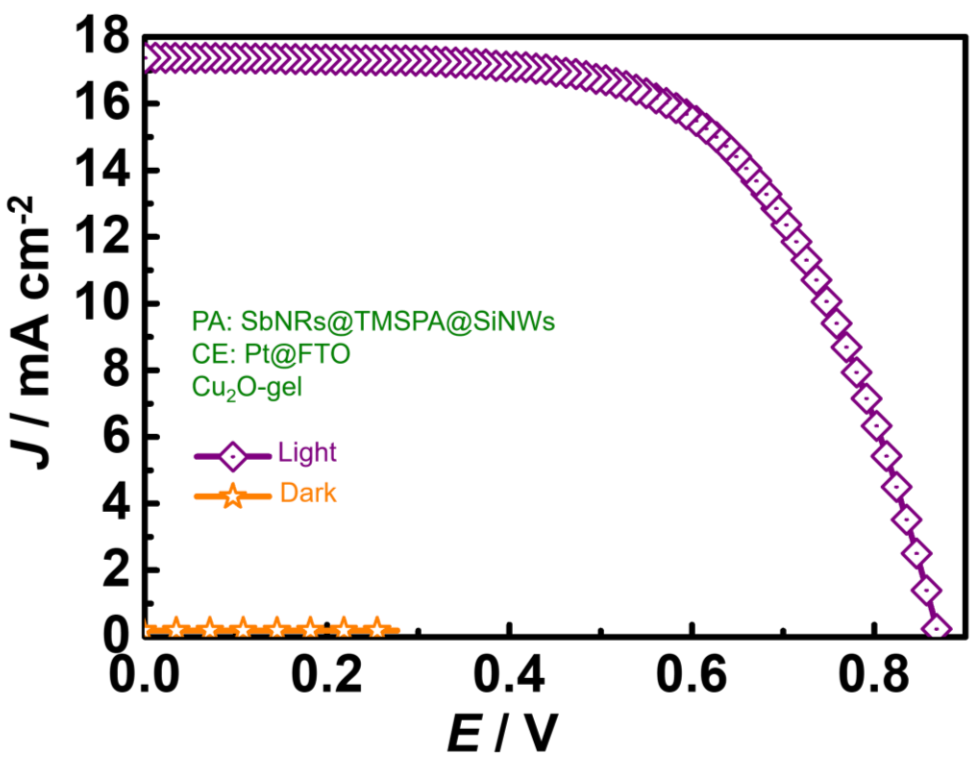

Figure S13 J-V plot of SbNRs@TMSPA@SiNWs/Cu_2O-gel/Pt@FTO under AM1.5 1 sun illumination.

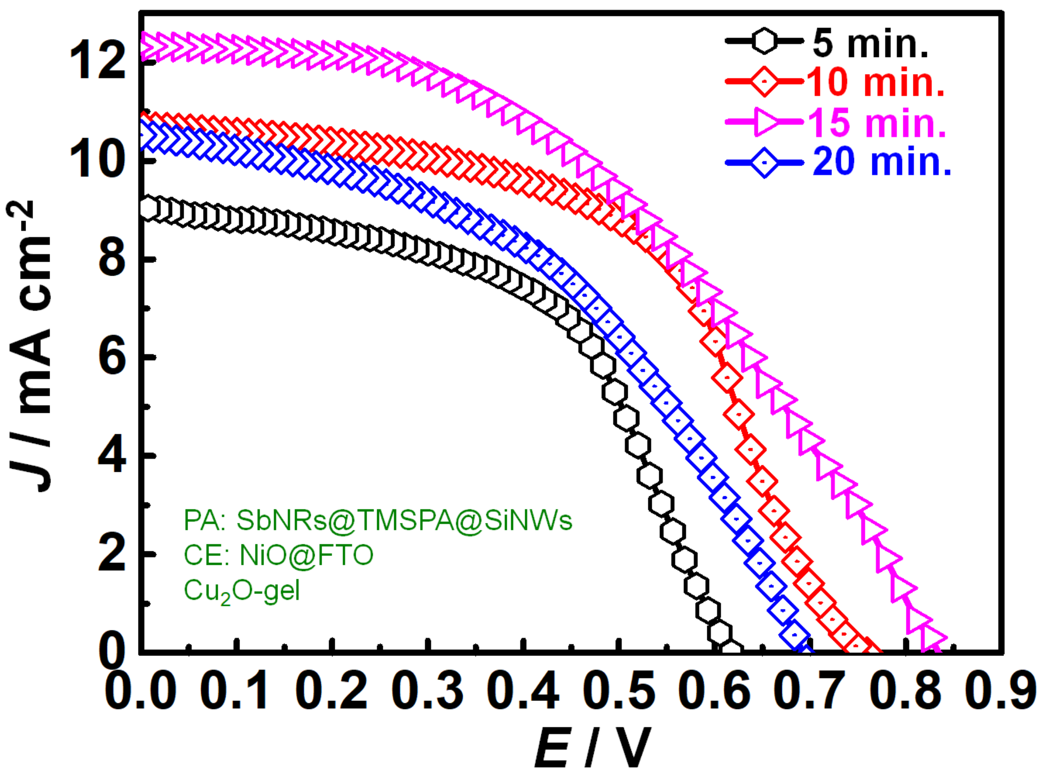

Figure S14 J-V curves of SbNRs@TMSPA@SiNWs (x min.)/Cu_2O-gel/NiO@FTO cells where x = 5, 10, 15 and 20 min. and x is the etching time for Si wafer.

Table S7 PV parameters of SbNRs@TMSPA@SiNWs (x min.)/Cu_2O-gel/NiO@FTO cells where x = 5, 10, 15 and 20 min. and x is the etching time for Si wafer.

Time (min.)	V_{OC} (mV)	J_{SC} ($mAcm^{-2}$)	FF	PCE (%)
5	617	9.1	0.54	3.05
10	759	10.7	0.55	4.46
15	841	12.3	0.45	4.67
20	708	10.5	0.46	3.39

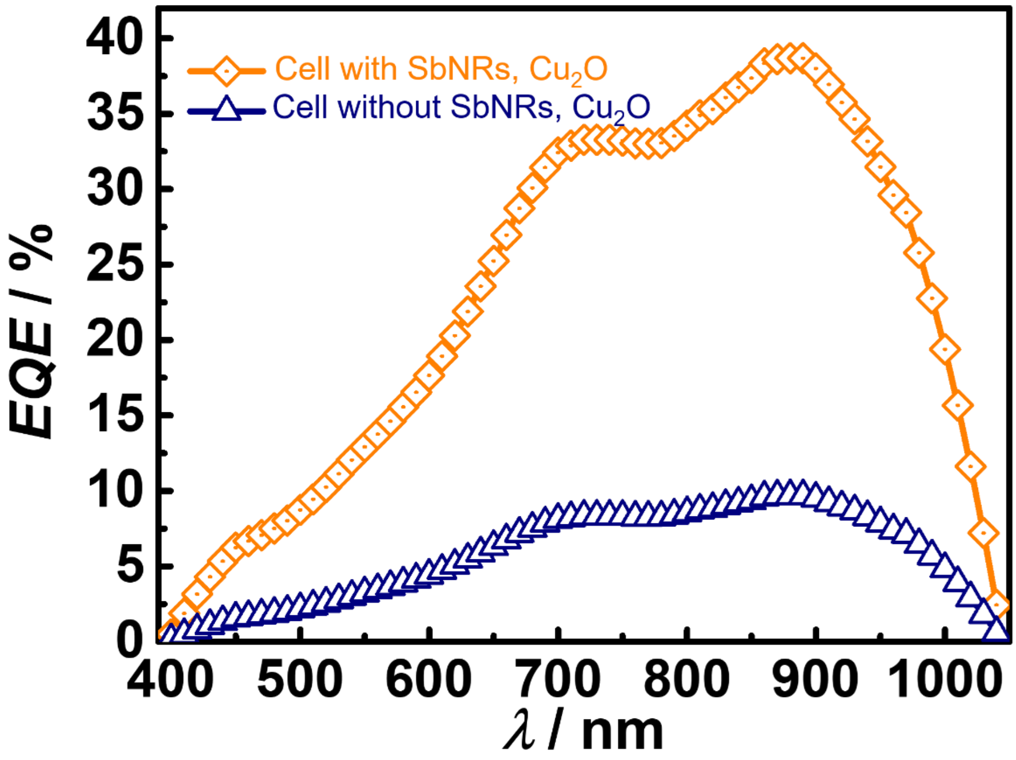

Figure S15 EQE spectra of SbNRs@TMSPA@SiNWs/Cu_2O-gel/NiO@FTO and TMSPA@SiNWs/gel/NiO@FTO cells.

Table S8 Stability parameters of SbNRs@TMSPA@SiNWs/Cu_2O-gel/NiO@FTO for 500 hours.

Time (h)	V_{OC} (mV)	J_{SC} (mA cm^{-2})	FF	PCE (%)
0	771	12.92	0.72	7.17
20	763	12.18	0.70	6.50
40	755	11.89	0.68	6.10
60	753	11.68	0.65	5.72
80	744	11.38	0.62	5.25
100	738	11.25	0.59	4.90
120	735	11.23	0.57	4.71
140	731	11.16	0.52	4.24
160	726	11.09	0.52	4.19
180	723	11.07	0.50	4.0
200	720	11.02	0.47	3.73
220	716	10.87	0.44	3.42
240	710	10.72	0.41	3.12
260	702	10.64	0.41	3.06
280	680	10.33	0.40	2.81
300	675	10.24	0.39	2.69
320	660	10.18	0.38	2.55
340	655	10.12	0.37	2.45
360	645	10.02	0.36	2.33
380	640	9.87	0.36	2.27
400	633	9.82	0.35	2.17
420	628	9.72	0.33	2.01
440	625	9.63	0.32	1.93
460	624	9.59	0.31	1.85
480	620	9.56	0.30	1.78
500	616	9.56	0.30	1.77

Table S9 Stability of different solar cells, under extended illumination.

Effect of SbNRs: SbNRs@TMSPA@SiNWs/Cu_2O-gel/NiO@FTO Cell				
Time (h)	V_{OC} (mV)	J_{SC} (mA cm^{-2})	FF	PCE (%)
0	840	12.3	0.46	4.75
100	828	12.13	0.45	4.51
200	820	12.05	0.44	4.35
300	803	11.99	0.41	3.95
400	794	11.63	0.40	3.69
500	780	11.45	0.40	3.59
Effect of TMSPA: TMSPA@SiNWs/Cu_2O-gel/NiO@FTO Cell				
0	629	7.5	0.58	2.74
100	613	7.3	0.51	2.27
200	594	7.04	0.43	1.80
300	542	6.63	0.39	1.40
400	503	6.27	0.33	1.04
500	472	5.81	0.30	0.82
Cell without TMSPA: SiNWs/Cu_2O-gel/NiO@FTO				
0	651	8.1	0.53	2.8
100	621	7.4	0.45	2.06
200	592	6.25	0.39	1.44
300	440	4.82	0.35	0.74
400	275	3.6	0.29	0.28
500	156	2.3	0.25	0.09

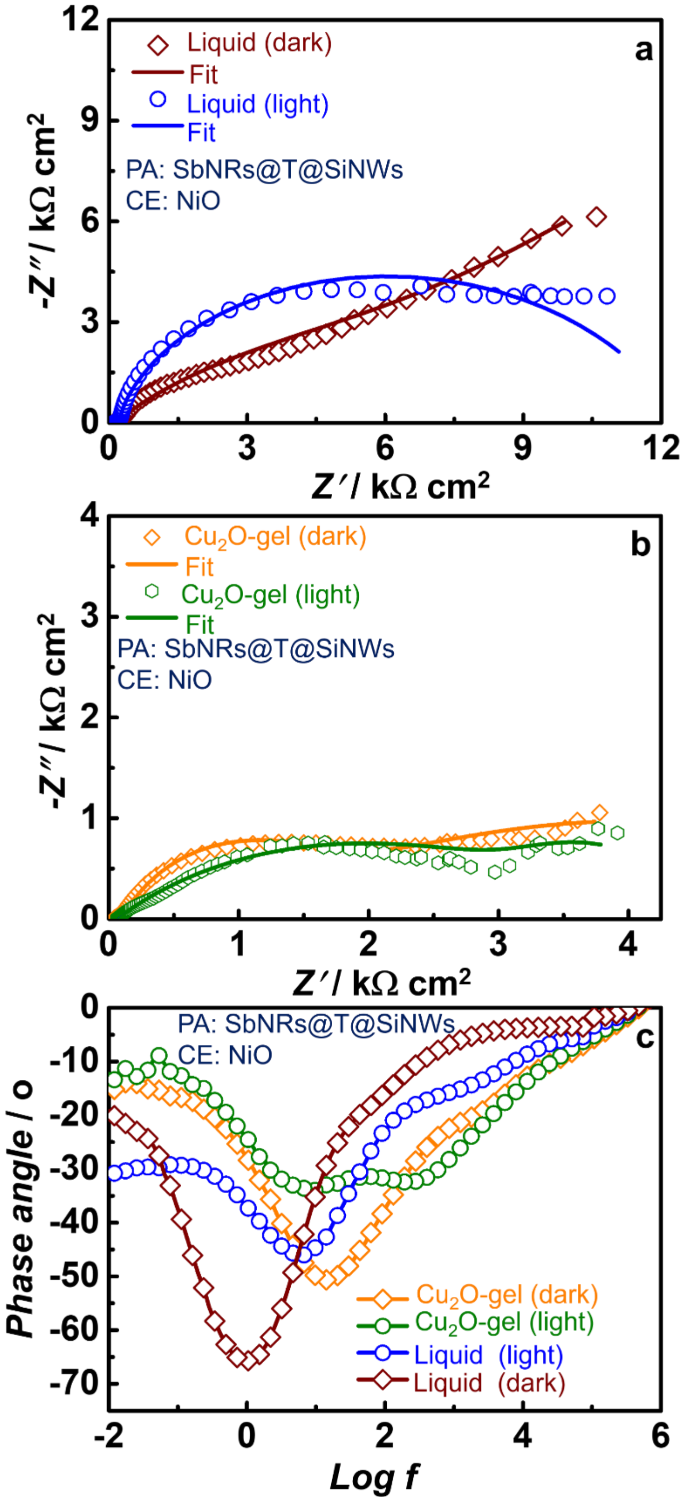

Figure S16 Nyquist plots of SbNRs@TMSPA@SiNWs/electrolyte/NiO cells: (a) liquid, (b) Cu_2O-gel and (c) Bode plots corresponding to (a) and (b).

Table S10 Nyquist fitted parameters of the solar cells both under dark and light.

Cells (condition)	R_b (Ω cm^2)	R_{ct} (Ω cm^2)	R_{rec} (Ω cm^2)	Y_{o1} ($\mu\Omega^{-1}$)	N	Y_{02} ($\mu\Omega^{-1}$)	N	χ^2	Circuit
SbNRs@TMSPA@SiNWs/liquid/NiO (light)	128	185	12366	12.1	0.98	20.1	0.68	0.002	R(QR)(QR)
SbNRs@TMSPA@SiNWs/liquid/NiO (dark)	127	5386	-------	42	0.80	------	-----	0.009	R(RQ)W
SbNRs@TMSPA@SiNWs/Cu_2O-gel/NiO@FTO (light)	59	884	3650	4459	0.98	38.58	0.49	0.001	R(QR)(QR)
SbNRs@TMSPA@SiNWs/Cu_2O-gel/NiO@FTO (dark)	56	1125	5855	18.3	0.80	163	0.8	0.001	R(QR)(QR)
